# Synthesis and translation of research and innovations from polio eradication (STRIPE): initial findings from a global mixed methods study

**DOI:** 10.1186/s12889-020-09156-9

**Published:** 2020-08-12

**Authors:** Olakunle Alonge, Abigail H. Neel, Anna Kalbarczyk, Michael A. Peters, Yodi Mahendradhata, Malabika Sarker, Eme Owoaje, Wakgari Deressa, Patrick Kayembe, Ahmad Shah Salehi, S. D. Gupta

**Affiliations:** 1 Johns Hopkins Bloomberg School of Public Health, 615 N. Wolfe St, Baltimore, MD 21205 USA; 2 Universitas Gadjah Mada, Faculty of Medicine, Public Health and Nursing, Yogyakarta, Indonesia; 3 BRAC James P. Grant School of Public Health, BRAC University, Dhaka, Bangladesh; 4 Heidelberg Global Institute of Health (HIGH), Heidelberg University, Heidelberg, Germany; 5 University of Ibadan College of Medicine, Ibadan, Nigeria; 6 Addis Ababa University School of Public Health, Addis Ababa, Ethiopia; 7 University of Kinshasa School of Public Health, Kinshasa, Democratic Republic of Congo; 8 Global Innovations Consultancy, Kabul, Afghanistan; 9 Indian Institute of Health Management Research, Jaipur, India

**Keywords:** Polio, Global Polio Eradication Initiative, GPEI, Implementation, Knowledge translation

## Abstract

**Background:**

Lessons from polio eradication efforts and the Global Polio Eradication Initiative (GPEI) are useful for improving health service delivery and outcomes globally. The Synthesis and Translation of Research and Innovations from Polio Eradication (STRIPE) is a multi-phase project which aims to map, package and disseminate knowledge from polio eradication initiatives as academic and training programs. This paper discusses initial findings from the knowledge mapping around polio eradication activities across a multi-country context.

**Methods:**

The knowledge mapping phase (January 2018 – December 2019) encompassed four research activities (scoping review, survey, key informant interviews (KIIs), health system analyses). This paper utilized a sequential mixed method design combining data from the survey and KIIs. The survey included individuals involved in polio eradication between 1988 and 2019, and described the contexts, implementation strategies, intended and unintended outcomes of polio eradication activities across levels. KIIs were conducted among a nested sample in seven countries (Afghanistan, Bangladesh, the Democratic Republic of Congo, Ethiopia, India, Indonesia, Nigeria) and at the global level to further explore these domains.

**Results:**

The survey generated 3955 unique responses, mainly sub-national actors representing experience in over 74 countries; 194 KIIs were conducted. External factors including social, political, and economic factors were the most frequently cited barriers to eradication, followed by the process of implementing activities, including program execution, planning, monitoring, and stakeholder engagement. Key informants described common strategies for addressing these barriers, e.g. generating political will, engaging communities, capacity-building in planning and measurement, and adapting delivery strategies. The polio program positively affected health systems by investing in system structures and governance, however, long-term effects have been mixed as some countries have struggled to institutionalize program assets.

**Conclusion:**

Understanding the implementing context is critical for identifying threats and opportunities to global health programs. Common implementation strategies emerged across countries; however, these strategies were only effective where organizational and individual capacity were sufficient, and where strategies were appropriately tailored to the sociopolitical context. To maximize gains, readiness assessments at different levels should predate future global health programs and initiatives should consider system integration earlier to ensure program institutionalization and minimize system distortions.

## Background

Between 1988 and 2017, over 15 billion US dollars have been spent towards efforts to eradicate polio globally [1]. As the Global Polio Eradication Initiative (GPEI) enters its final stretch, public goods (infrastructure, systems, and knowledge assets^1^) created by this initiative, which have contributed to a decrease in the global incidence of polio by 99% and which are relevant for advancing population health, will be lost if they are not clearly articulated and effectively disseminated or repurposed. This is against the backdrop that these public goods were developed in some of the most disadvantaged regions in the world, including many low-and middle-income countries (LMICs) with weak health systems grappling with the double burden of communicable and non-communicable diseases. Widespread uptake of key public health lessons, based on both positive and negative experiences, do not occur passively but require active strategies to package and communicate such lessons to potential adopters. Similarly, without active strategies knowledge assets from the GPEI may not realize their potential of facilitating delivery of life-saving programs and strengthening health systems for vulnerable populations. These include strategies for mapping, packaging, and disseminating relevant knowledge products to various target audiences.

Several efforts have been made to document the lessons learned from the GPEI and polio eradication activities broadly (e.g. GPEI transition documentation in different countries, Centers for Disease Control and Prevention (CDC) journal supplements on lessons learned from GPEI, CDC archives and oral history project, United States Agency for International Development (USAID)/Collaborations and Resources (CORE) group polio project journal supplements and conferences, USAID work with the Communication Initiative, and others). These efforts have generated lessons learned in peer reviewed journals stemming from the perspective of a single country/region/stakeholder (e.g. India [2] or the South-East Asia Region [3]), within a single organization or entity (e.g. Stop Transmission of Polio (STOP) program [4], Rotary [5], CORE group [6], or the Immunization Systems Management Group [7]), or within a single stream of GPEI activities (e.g. the switch to bivalent vaccine [8], social engagement and communication interventions [9]). Findings from these efforts can be categorized under three broad categories based on their primary objectives and the facet of knowledge that they prioritized: [1] those that focused on describing the best practices and success stories of the GPEI [7], [2] those that focused on the failures and missed opportunities to achieve the goal of eradicating polio much sooner [2], and [3] those that described the impact of the GPEI on health systems through transition planning [10].

Only a few of these efforts, however, have described a comprehensive and systematic approach to capture the multifaceted knowledge surrounding global polio eradication from a multilevel perspective (global, national, and sub-national), or described any active strategies to facilitate the uptake of these lessons by potential adopters. Gaps exist in these efforts, primarily in that the methods for generating lessons learned are generally not described, and methods that are documented do not include active data collection. Instead, lessons learned have typically been generated by experts in a top-down manner without major input from individuals involved in program implementation at the field level (excepting the CORE group project [6]). Finally, while some efforts are specifically focused on applying lessons learned to future lifesaving programs [11], most of the literature is focused on the application to other eradication programs, such as transitioning resources and experiences to measles and rubella eradication. The vast array of activities that make up the GPEI have far-reaching applications in public health programming and health systems strengthening in resource-constrained settings. Thus, there is a need to systematically synthesize lessons learned from different levels of the polio eradication experience across geographies, organizations and work streams and apply them to future lifesaving initiatives.

**Fig. 1:**
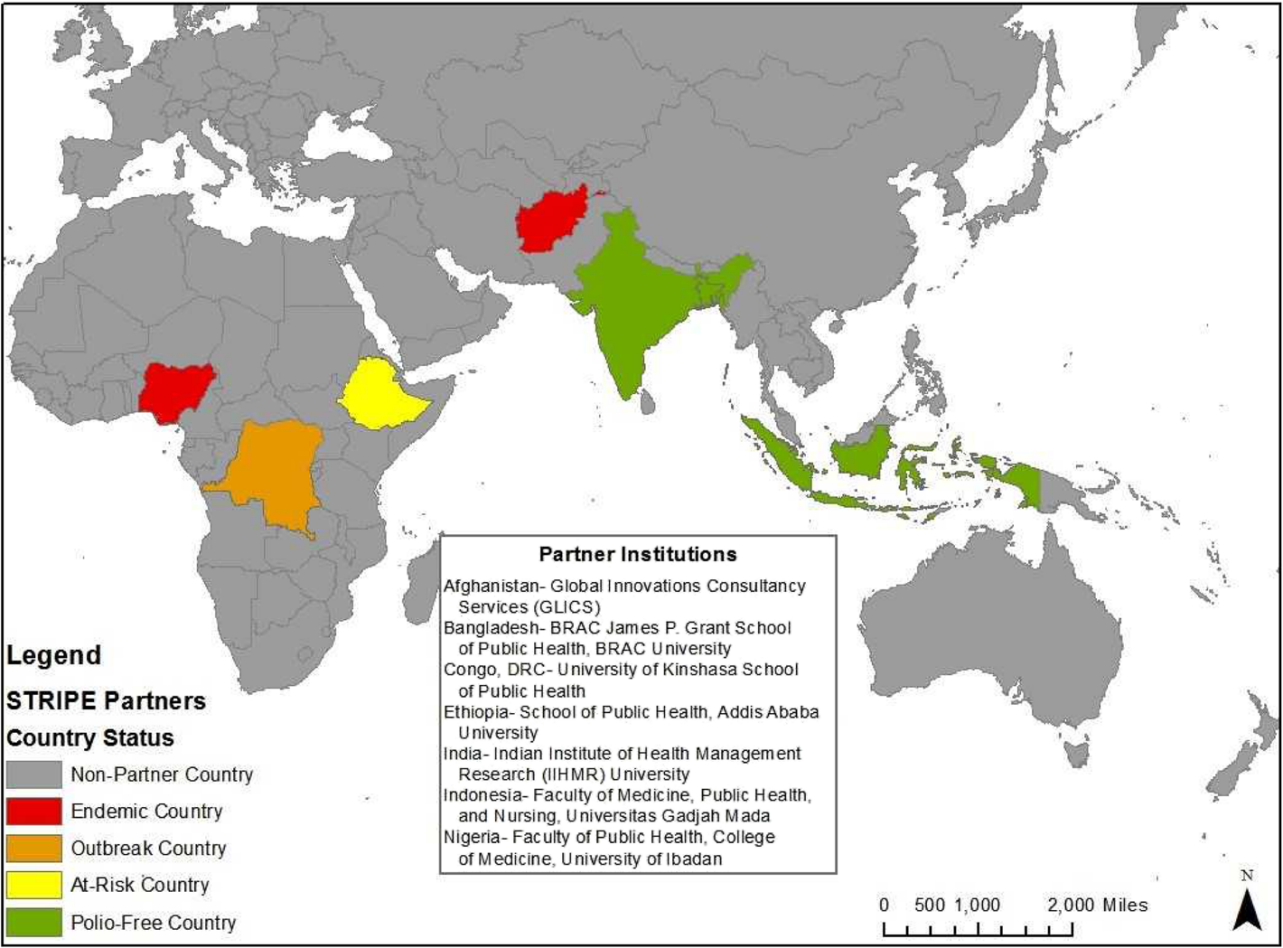
Focus countries for primary data collection. Source: Authors prepared a map of country partners in ArcGIS Desktop.

The Johns Hopkins Bloomberg School of Public Health (JHSPH), in conjunction with a consortium of international academic partners, is working to map, package and disseminate knowledge assets under various polio eradication initiatives, including the GPEI. Products of this research include academic and training programs for various global health audiences which will facilitate effective implementation of lifesaving programs globally and contribute to the legacy of the GPEI in advancing global health beyond polio eradication. This project, also known as the Synthesis and Translation of Research and Innovations from Polio Eradication (STRIPE) project, selected a consortium of seven institutional partners across wide-ranging contexts in which GPEI activities occurred. Knowledge is inherently linked to the context from which it is derived [12] and it is impossible to capture the rich and varying experiences of GPEI from a single country’s perspective. To incorporate the plurality of contexts where polio eradication activities have been implemented, the STRIPE project included partners from focus countries representing various GPEI context typologies, that is, at least one country under each of the epidemiological classifications for polio (endemic, outbreak, at-risk and polio-free) [13] (see Fig. 1).^2^ These focus countries include Afghanistan, Bangladesh, the Democratic Republic of Congo (DRC), Ethiopia, India, Indonesia, and Nigeria. The focus countries were further selected to represent different geographical regions where polio eradication activities have intensified in recent times, country income classifications, conflict-affected compared to stable countries, and countries that could serve as influential regional leaders to facilitate uptake of knowledge by other countries in their region.

In addition, a high-level technical advisory committee (TAC) was formed to advise on the different steps in the project, assist in gaining access to relevant polio program data, conduct internal reviews on key knowledge products, and provide access to high-level global stakeholders. The TAC includes individuals representing GPEI core partners (the World Health Organization (WHO), United Nations International Children’s Fund (UNICEF), Centers for Disease Control and Prevention (CDC), the Bill and Melinda Gates Foundation (BMGF), and Rotary International), ministries of health in LMICs, non-governmental organizations (NGOs), faculty from schools of public health in LMICs and high-income countries, and other relevant agencies.

The STRIPE project involves four key phases: knowledge mapping, synthesis, packaging, and dissemination and uptake. This paper describes two of the main methods under the knowledge mapping activities, the survey and key informant interviews, and presents initial findings drawn across global, national and sub-national levels. It is hoped that these descriptions are useful for facilitating the effective implementation of other global health programs such as those addressing the prevention and control of non-communicable diseases, neglected tropical diseases, and other vaccine-preventable diseases, and those focused on strengthening health systems. It is also hoped that the methods described are useful for knowledge translation efforts of lessons from other public health programs globally.

## Methods

The knowledge mapping activities utilized a sequential explanatory mixed methods design [14, 15]. A tacit knowledge survey was conducted at the global level, and at national and sub-national levels in all focus countries between August 2018 and December 2019, with the global survey remaining open through April 2019. The survey collected information on contextual factors that were key barriers and facilitators of the polio eradication activities, and the level where barriers originated from (global, national, sub-national).

Respondents for the survey were defined as any living individuals 18 years or older who have been involved in implementing polio eradication activities (including research, funding, strategy and practice-related activities) for 12 or more continuous months between 1988 and 2019. At the global level, these individuals were systematically identified through various working groups, committees, research networks, and communities of practice among the core GPEI partners (including WHO, UNICEF, CDC, BMGF, and Rotary), bilateral agencies such as USAID, NGOs within the CORE group, and other global government officials and donors. Survey recruitment was extended to actors across operational levels within each organization. Similarly, institutional partners in each of the seven focus countries defined a polio universe of actors at the national and sub-national levels to include relevant polio or immunization personnel and managers at the various levels of the health system, with a particular focus on including frontline field workers and healthcare providers at community-level facilities. This process is outlined in more detail in another paper in this series [16].

The survey tool (Additional file 1) was developed based on the Consolidated Framework for Implementation Research (CFIR) [17], the Organizational Social Context framework [18], and the socioecological model [19]. It covered key constructs describing the internal and external contexts for implementation, implementation strategies, intended and unintended consequences, and other descriptors including polio eradication activities, type of organizations, and demographic information related to the respondents’ role in polio eradication activities at different socioecological levels. The survey was self-completed and implemented online using the Qualtrics electronic data platform [20]. The survey was back translated from English to Bahasa-Indonesia, Bangla, Dari, French, Hindi, Spanish, and Urdu languages. Supplemental face-to-face and phone surveys were conducted by interviewers at national and sub-national levels in areas of some focus countries where electronic data capture was infeasible. Interviewers were university graduates who received 2–4 days training on the survey instruments and data collection approaches and were supervised by project personnel who also conducted random reassessment of data collected by the interviewers for accuracy.

The survey data was extracted and cleaned in R (version 3.3.2) [21] and descriptive analysis was done to describe the polio eradication activities, internal and external contextual factors affecting the implementation of these activities, and whether these factors were facilitators or barriers of the eradication activities. All quantitative data analyses were conducted in STATA I/C (version 14) [22].

Key informant interviews were administered to a nested sample of survey respondents to further explore implementation challenges of polio eradication activities, strategies for addressing these challenges, and their intended and unintended outcomes across the levels of the socioecological framework [19]. To determine the nested sample, survey responses were reviewed to identify individuals who highlighted challenges faced as part of polio eradication activities that were relevant to other health services delivery areas, and representative of the variety of challenges mentioned. Respondents were further prioritized to ensure representativeness across levels of the health system, geography, organizations, and areas of expertise.

The KII interview guide (Additional file 2) prioritized questions on polio program organization and change over time, contextual challenges (internal and external), strategies utilized to address challenges faced, and key lessons learned. The KIIs were conducted by two interviewers with expertise in qualitative research at the global level, and 3–4 interviewers within each focus country. The interviewers at the country-level were university graduates with prior experience conducting qualitative interviews, trained on the interviewer guide via institutionally determined processes in each country, typically a 2 or 3-day training. A training manual on qualitative data collection was also developed and reviewed with research leads to ensure standard interview processes were met, i.e. processes for recruitment, transcription, memo drafting, and data management. The interviews were conducted in local or official languages in each country between February 2019 and May 2019 and the data was transcribed and translated into English for analysis. A codebook was developed according to the CFIR [17] and SEM [19] models to capture key lessons learned from the polio eradication experience.

Coding and analysis were conducted in Dedoose (version 8.2.31) [23]. Four reviewers conducted a pilot test on two interviews from two different countries. Each reviewer applied the codebook to both interviews. Analytics were run in Dedoose to detect any differences between reviewers and a meeting was held to review this data and reach consensus on the use of codes moving forward. Post-analysis data validation was also conducted, including cross-checking key findings with respondents.

The quantitative and qualitative data were mixed such that the quantitative survey data was used to describe the frequency of major barriers to polio program activities organized along domains from CFIR (which includes characteristics of the program, implementer, and contexts), and the KII data was used to explain further how these barriers played out in different contexts and the strategies that were used to address barriers.

The strength of the STRIPE study includes the use of a mixed method approach and representation of multiple context typologies in the primary data collection. The systematic approach for conducting the quantitative survey, and the large sample size of survey respondents also allow for more valid and robust conclusions. Unlike similar lessons learned efforts from global polio eradication activities, findings from the STRIPE study are not based exclusively on literature review and expert consultation with global leaders, but are also based on primary data collected mostly at national sub-national levels, which provides more insight of implementation challenges faced by managers and frontline health workers across two continents. The survey response rate was between 6.8% (Ethiopia) and 34.75% (DRC) which overlapped the expected range for typical online-based survey [24]. However, the study is not without limitations and the data from which the findings are derived is subject to recall bias. Though reasonable efforts were made to minimize bias via data triangulation and reviews, survey respondents may have only recalled more recent challenges or strategies, they may also have only recalled discussions around issues that resonated personally with them and not necessarily based on an objective assessment of the implementation process. Notably, our analysis prioritizes the experience of those directly involved in implementing polio eradication activities. As a result, some perspectives may be missing from our analysis, namely public health workers who were impacted by but not directly involved in polio eradication, as well as community members within our study countries. This study also presented a limited view of the challenges and strategies from the global level. However, most similar studies have emphasized experiences from the global level [2–9], and this paper provides a wholistic perspective, leveraging knowledge from actors across levels within global institutions, and filling a critical gap in the literature around lessons learned from the global polio eradication effort from the national and sub-national levels.

## Results

The survey included 3955 unique respondents across the global and country-level surveys. 296 (7.5%) individuals did not complete the survey and those entries were excluded from the analyses. The sample characteristics in Table 1 reflect the multiplicity of roles held by 3659 respondents that completed the surveys. The sample represents work experience in over 74 countries, with the highest preponderance of experience in the African region (61.4%, *n* = 2241), followed by the Southeast Asian region (26.4%, *n* = 971) and the Eastern Mediterranean region (20.3%, *n* = 744). Across surveys, the average number of years worked in polio eradication was 9.28 years. The respondents’ experience was somewhat concentrated between the years 2000 and 2019. While gender was excluded from the survey to ensure anonymity, among the KII respondents 74.5% were male and 25.5% were female. A majority of respondents for both the surveys and KII had worked at the sub-national level (state, district or sub-district level), and the highest number worked for government agencies (42.8%, *n* = 3657 and 40.7%, *n* = 81 for the survey and KIIs respondents, respectively), followed by GPEI partners (WHO, UNICEF, CDC, BMGF and Rotary International), and other implementing or non-governmental organizations. Many survey respondents were directly involved in core program functions, e.g. vaccination (27.5%, *n* = 2097) and surveillance (18.2%, *n* = 1389), while others were involved in supporting functions, e.g. community engagement (15.2%, *n* = 1160) and monitoring and evaluation (13.1%, *n* = 997). Fewer respondents indicated involvement in upstream activities, e.g. strategy development (7.4%, *n* = 565), partnership development (4.0%, *n* = 303), and resource mobilization (2.8%, *n* = 211).

**Table 1:** Characteristics of Respondents

	Survey (*N* = 3659) n (%)^a^	KIIs (*N* = 194) n (%)
**Country distribution**		
Global^1^	796 (21.8%)	18 (9.3%)
Afghanistan	513 (14.0%)	28 (14.4%)
Bangladesh	106 (2.9%)	17 (8.8%)
Democratic Republic of Congo	499 (13.6%)	23 (11.9%)
Ethiopia	101 (2.8%)	30 (15.4%)
India	401 (11.0%)	25 (12.9%)
Indonesia	322 (8.8%)	26 (13.4%)
Nigeria	921 (25.2%)	27 (13.9%)
**Levels worked**		
Global	565 (9.2%)	18 (9.3%)
National	894 (14.6%)	84 (43.3%)
State/District	3333 (52.7%)	70 (36.1%)
Sub-district/Frontline	1445 (23.5%)	22 (11.3%)
**Organizational representation**		
GPEI partners	2948 (34.5%)	61 (31.4%)
Government	3657 (42.8%)	96 (49.5%)
Implementing organizations	1600 (18.7%)	23 (11.9%)
Research organizations	119 (1.4%)	5 (2.6%)
Other	216 (2.5%)	9 (4.6%)
**Polio program goal**^**2**^		
Resource mobilization	213 (2.7%)	–
Partnership development	312 (4.0%)	–
Strategy development	577 (7.4%)	–
Strengthening delivery systems	926 (11.9%)	–
Vaccine administration	2132 (27.4%)	–
Surveillance	1426 (18.3%)	–
Community Engagement	1176 (15.1%)	–
Monitoring and Evaluation	1027 (13.2%)	–

^a^*Survey respondents were able to select multiple responses for the following characteristics:* ***the levels where they worked, their organizational representation, and the polio program goal*** *over the period 1988–2019****.*** *Hence, the sum of responses (n) under each of these characteristics is greater than the total number of respondents (N = 3659 for the survey)*

^1^*Global respondents refer to those respondents contacted by the global survey, as compared to surveys conducted in the seven focus countries. This sample therefore includes individuals who may have primarily worked at the global level but also supported polio eradication in one or multiple countries*

^2^*Survey respondents were asked to indicate polio program goals which they were involved as part of the survey response; this information was not gathered during key informant interviews, though respondents’ experiences were reviewed* a priori *to ensure representativeness across program goals*

### Implementation barriers and strategies for addressing them

#### External factors

External factors were the most frequently cited barriers to implementation in the survey (39.3% of all barriers) (Table 2). Among these, social factors accounted for the majority (44.3%) of all external barriers. The importance of the social environment to program success was echoed in the key informant interviews. The most commonly cited social factor in the KIIs was low vaccine demand, broadly defined to encompass a range of factors affecting vaccine uptake, an issue which manifested at both the individual and community levels to hinder the success of the polio program. Social factors posed a challenge across countries but were often confined to specific locales or communities.

**Table 2:** Barriers to Polio Program Success

CFIR Domain	Barrier Definition	Illustrative examples	All survey responses, ***N*** = 9714 n1 (% of N)^a^ *n2 (% of n1)*^b^
**External Factors**	**Political, economic, social, technological, legal, and other environmental factors**		**3826 (39.4%)**
Social	Communities are non-accepting and/or resistant to the intervention	• Vaccine hesitancy • Community fatigue given repeated campaigns, misaligned priorities • Lack of information	*1695 (44.3%)*
Economic	Insufficient revenue sources	• Limited economic resources	*1170 (30.6%)*
Political	Policymaker disinterest or resistance, limited windows of opportunity within the political climate, political structure non-conducive to coordinated action	• Low political will • Insecurity and conflict	*1115 (29.1%)*
Technological	Slow or limited advances of technologies used in implementing program activities	• Technological and infrastructural challenges affecting vaccine supply and surveillance	*626 (16.4%)*
Other	Challenges related to physical and human geography	• Geographical inaccessibility • Population migration	*817 (21.4.0%)*
**Process of activities**	**How activities were implemented**		**2144 (22.1%)**
Executing	Failing to carry out activities according to plan	• Lack of accountability mechanisms • Environmental disruptions to program implementation • IPV supply challenges	*1213 (56.6%)*
Engaging	Difficulty attracting and involving appropriate stakeholders in implementation	• Difficulty identifying appropriate stakeholders to engage given diverse administrative structures, cultural norms • Community mistrust	*915 (42.7%)*
Reflecting & Evaluating	Difficulty monitoring program progress and quality, including lack of regular debriefing about progress and experience	• Lack of supervision • Lack of formal processes for analyzing monitoring data and adapting plans accordingly	*803 (37.5%)*
Planning	Implementation schemes/methods not planned in advance, or poor quality of such methods	• Poor quality enumeration • Difficulty in planning large-scale changes, e.g. the switch from tOPV to bOPV	*758 (35.4%)*
**Characteristics of individuals**	**Characteristics of individuals within an organization involved in polio eradication activities**		**1773 (18.3%)**
Knowledge	Knowledge and beliefs about the activity - individuals did not have positive attitude toward the program, were unfamiliar with facts, truths and principles related to the intervention	• Misconceptions about the vaccine and its effects • Lack of awareness of vaccine benefits	*1121 (63.2%)*
Stage of Change	How likely (or not) the individual is to provide skilled, enthusiastic and sustained support of the program throughout the different stages of implementation	• Health worker fatigue resulting from campaign/vaccine fatigue from the communities	*566 (31.9%)*
Perception of organization	Poor perception of the organization and degree of commitment to the organization	• Temporary status of some frontline workers affecting commitment to organizational goal	*419 (23.6%)*
Self-efficacy	Lack of belief in one’s own abilities to execute required courses of action	• Health workers’ lack of understanding of the program, what’s expected of them	*394 (22.2%)*
**Organizational characteristics**	**Factors related to the organization(s) supporting implementation**		**1076 (11.1%)**
Structure	The age, social architecture, and size of an organization led to challenges	• Shifting structure of global partnership • Understaffing and shifting roles of staff	*236 (21.9%)*
Networks	The nature and quality of formal and informal communication within an organization led to challenges	• Limited communication channels between extension workers, program leads • Challenges related to dissemination of strategy from central to peripheral level, including securing buy-in	*439 (40.8%)*
Culture	The norms, values, and operating assumptions of an organization led to challenges	• Priorities dictated by managers • Limited voice given to field workers to propose adaptations	*349 (32.4%)*
Implementation Climate	Limited capacity for change, the receptivity of the team to the proposed intervention, the relative priority of project, organizational goals, incentive and rewards, etc. led to challenges	• Lack of consensus on program strategy • Waning prioritization of polio among some stakeholders	*398 (37.0%)*
Implementation Readiness	Lack of leadership engagement, limited available resources and poor access to knowledge and information led to challenges	• IPV shortage • Chronic underfunding of the health system	*469 (43.6%)*
**Program characteristics**	**Activities conducted to enable implementation, including technologies adopted**		**895 (9.2%)**
Intervention Source	Perception of whether the intervention was developed internally or externally led to challenges	• Imbalance between global and national priorities • Community distrust of western intervention	*276 (30.8%)*
Evidence	Perception of the quality and validity of the evidence did not support belief that the intervention would have the desired outcomes	• Concerns about relative effectiveness of OPV and IPV	*302 (33.7%)*
Relative Advantage	Perception that there was another, better approach	• Concern that polio program is run in parallel to (and at expense of) routine immunization	*200 (22.3%)*
Adaptability	The activity was not adapted, tailored or refined to meet local needs	• Lack of understanding of community norms to guide adaptation of implementation activities	*361 (40.3%)*
Trialability	No ability to test on a small scale and reverse course if warranted	• Perception of polio program as too big to fail even in the face of coordination and implementation failure affecting certain activities	*101 (11.3%)*
Complexity	Perceived difficulty of implementation reflected by its duration, scope, radicalness, disruptiveness, centrality, intricacy, and number of steps required	• Difficulty sustaining the cold chain in hard-to-reach areas • Health worker and community fatigue	*284 (31.7%)*
Design Quality & Packaging	Difficulty arising from how the intervention is bundled, presented, and assembled	• Challenges related to use of injectable vaccine (IPV) • Vaccine wastage due to how IPV and OPV were packaged, especially in hard-to-reach areas	*162 (18.1%)*
Cost	Cost of intervention and its implementation, including investment, supply, and opportunity costs	• Difficulty financing program functions previously supported by donors • High cost of implementation in hard-to-reach areas	*252 (28.2%)*

^a^*Each respondent was allowed to choose all relevant domains that contributed as barriers to polio program goals. Hence, the sum of all responses, n1 (9,714) is greater than sample size for all survey respondents (3659)*

^b^*Within each domain, respondents were similarly allowed to choose all relevant categories that contributed as barriers to polio program goals,* e.g. *for the external factor domain, each respondent selected multiple categories under that domain such that the sum of all category-specific responses (n2) is greater than n1 (3,826) for that domain*

KII respondents described the need to address low levels of awareness of immunization services and its benefits, particularly among communities which lived outside the formal system, and cited vaccine hesitancy (defined in the literature as delay in acceptance or refusal of vaccination despite availability of vaccination services [25]) as a major reason for low vaccine demand and one of the biggest threats to program success. In some places, vaccine hesitancy was due to a general mistrust of government programs based on historical antecedence and this affected vaccination efforts more broadly, including refusal of non-polio vaccines. In other places, the vaccine hesitancy was specific to the uptake of the Oral Polio Vaccine (OPV) due to fatigue and concern over repeated campaigns and house-to-house vaccination, and a perception of misalignment of health priorities by community stakeholders. Other manifestations of OPV hesitancy across different contexts included lack of awareness, concern over multiple doses, fear that vaccines could cause infertility, fear that vaccines contained HIV, and concern that vaccines were ‘non-halal’ in some communities. The duration of the polio program also contributed to waning program acceptance. As one respondent explained,

“*… there are families, they refuse taking vaccines, they do not agree with the program. This program has started in 1994 and there is one campaign every month … between 2011 and 2018 we almost had more than 10 campaigns every year, means we have knocked on the door of the household 10 times a year and vaccinated the children. Now the community is tired of vaccination and they want a change in the program. They request other things … clean drinking water, access to other health services, therefore the interest of the people has been decreased with the program.”* – Subnational actor, Afghanistan.

Strategies used to address these barriers were also targeted to the community and individual levels. At the community level, a key strategy was identifying and preparing champions and early adopters. Religious, community, and local leaders served as “gatekeepers” between implementers and the community who encouraged vaccination within their communities and thus facilitated program implementation. Increasing awareness of the population, including dispelling concerns about vaccination, also relied on individual level appeals via social mobilization activities. Across contexts, health workers and/or community volunteers were trained in information, education and communication (IEC) tactics and deployed as outreach staff to promote the polio program, and to follow-up with hesitant families as needed. This strategy was viewed as effective for increasing polio vaccine demand across countries, so long as both messengers and messages were tailored to the local context. Notably, in some countries these strategies were developed only after issues of vaccine hesitancy threatened the eradication goal and were not planned for at the outset of the initiative.

Economic and political factors were the next major external factors that were indicated as barriers to success from the survey, 30.6% and 29.2% respectively. Economic and political factors also emerged from the KIIs as salient for implementers because of the way these challenges contributed to other key barriers, and because of the relative effort required to address them. Economic challenges fell predominantly under two categories: economic deprivation among communities where polio eradication activities occurred, and low economic development leading to chronic underfunding of the health system. Economically deprived communities were suspicious of the polio community that emphasized polio eradication goals while basic livelihood issues were neglected, and this contributed to mistrust in these communities. In countries with low economic development, health system gaps as a result of chronic underfunding significantly impacted the execution of the polio program objectives. Political factors discussed by respondents included low political will and lack of polio program ownership in-country, political favoritism, and insecurity and conflict. These challenges operated at the policy, environmental, and community levels and varied considerably by country.

Regarding political will, key informants discussed the imbalance that was sometimes felt between global stakeholders who applied pressure to achieve eradication goals and national actors who were facing competing priorities. Respondents also discussed the difficulty in sustaining program support through election cycles, as well as generating support at multiple levels of the system in countries with devolved accountability structures. These issues impacted both program ownership and financing. Respondents in countries heavily dependent on donor support expressed concern that improvements to health system delivery generated by the polio program would deteriorate with waning donor funds, and without adequate institutionalization.

In conflict-affected and insecure areas, respondents recounted repeated disruptions to service delivery as a result of conflict and described lack of accessibility and concerns over health worker safety as persistent barriers to implementation. The impact of insecurity cannot be overstated; one global respondent suggested that inaccessibility due to political situations, complex emergencies, civil war, and armed conflict was “the single most important challenge” faced in the endgame stage of polio eradication, noting that even where the polio initiative succeeded in reaching the population it could negatively impact social acceptance. They explain,

“*situations like in Afghanistan and Pakistan or northern Nigeria … populations are extremely deprived populations for many, many years and severely conflict-affected, don’t get essential services, don’t even have clean water, and then if … the only service that reaches them is a polio vaccine, that could lead to a huge trust deficit.” – Global respondent.*

This suggests the compounding nature by which socio-political factors have impacted implementation of polio eradication activities and stymied progress toward achieving eradication.

To improve political will, GPEI partners advocated on behalf of the program and worked to involve stakeholders and workers in the implementation effort. Where these appeals were targeted varied given differences in administrative structures between countries. The usefulness of high-level advocacy was discussed in several contexts; in decentralized systems, these efforts were most effective where stakeholders at the sub-national level were also engaged recurrently, particularly where staff turnover occurred. Success of these strategies was somewhat mixed across contexts, but where political will was strong, it was a key facilitator of program success. One respondent explained the importance of the change in political will over time, saying:

“*… in between there was leadership in the ministry of health which were not so convinced so there were threats that the whole programme might collapse anytime, but there were groups which convinced that no, we should not give up, there were these massive surges of outbreak of polio and it was taken care well by the programme so the leadership, support and direction was very important.” -*National actor, India.

In fragile areas, implementers adapted service delivery tactics to account for insecurity, though this often meant waiting for the dynamics of the conflict to change as accessibility in conflict zones was infeasible. Where possible, vaccinators capitalized on “days of tranquility” to conduct mobile campaigns and conducted vaccinations in buffer zones and border crossing areas. Implementers also relied on satellite imagery and community informants to assess coverage gaps and identify cases. In Nigeria, military personnel were trained on cold chain management and vaccination and were able to deliver services directly; in other areas, however, implementers needed to be careful not to engage stakeholders who might politicize vaccination activities. In Afghanistan, for example, a respondent explained that,

*“we want to have the program as neutral as possible without any visible engagement of parties of conflict.” –* Global respondent.

#### Process of activities

In addition to these external factors, survey and KII respondents indicated a number of internal barriers to success which affected the polio program. 22.2% of barriers identified by the survey were barriers related to the process by which activities were implemented; of those process-related barriers, a majority (56.7%) fell under issues with executing, that is ability to carry out activities according to plan. From the KIIs, issues related to program execution, engagement, evaluation, and planning were all cited as common factors and included disruptions to program implementation and health system gaps. These challenges operated primarily at the organizational level, and though country capacity varied significantly, were experienced to some degree across all study countries.

Considering program execution, the KIIs suggest a strong link between external and internal barriers to implementation. Respondents repeatedly described how external factors (e.g. insecurity, lack of cooperation from some communities, low economic development) made it more challenging to implement activities as intended (i.e. issues related to program execution). Likewise, health systems gaps made it difficult to carry out activities according to plan. Human resource issues were the most often cited health systems gap across countries, including challenges ranging from health worker shortages, maldistribution, low pay, lack of supervision, and health worker fatigue. Other health systems challenges which required addressing were supply chain issues, e.g. OPV and IPV stockouts and lack of cold chain infrastructure, as well as lack of surveillance and laboratory capacity. These issues were exacerbated in remote, hard-to-reach areas that required additional human resources and posed significant logistical challenges. Describing the confluence of these issues one respondent said:

*“Look now, to do the polio vaccination, the routine vaccination or the campaign; we have said we have kebeles, those kebeles don’t even have refrigerators. Kebele may have had a [sic] health post but with no health workers … and trainings were not given to health workers who manage [sic] those refrigerators.”* – Subnational actor, Ethiopia.

Some of the strategies discussed for addressing external barriers including ongoing stakeholders’ engagement and political advocacy were also successfully applied to address issues related to executing the program. Other strategies to address these issues were multifaceted and included addressing issues around specific health system inputs, as well as improving management processes. One strategy commonly cited was assessing organizational ability and readiness in order to identify barriers that may impede implementation, as well as strengths that could be used in the implementation effort. This allowed implementers to optimize organizational structure, for example, right-sizing team composition with clear roles for the vaccinator, supervisor, and community leaders, and making real-time adjustments as needed, such as shifting the outreach plan where staffing was insufficient, or shifting vaccine supply between posts where stockouts were occurring in critical areas (as defined by polio program managers). Another key strategy was to alter incentive and disincentive structures for providers. Polio workers often received additional or higher pay, particularly for campaign work, and volunteer staff received in-kind incentives, although this strategy led to demotivation of health workers not involved in polio campaigns – a negative and unintended outcome. Conversely, new supervisory structures were put in place to ensure health workers’ accountability. Finally, significant investment was made to recruit, designate and train leaders across the functional areas of the program and health system. These strategies led to improved organizational capacity and health worker motivation which improved polio program execution; however, the sustainability of those gains was dependent on the degree of integration of polio structures within the broader health system and in some cases drew health workers away from routine service delivery.

Challenges related to program evaluating and reflecting (i.e. feedback and debriefing on implementation progress and quality), and planning were cited by somewhat fewer survey respondents. Per the KIIs, however, strategies deployed in these areas were some of the most effective facilitators of program activities, and thus contributed to addressing process barriers broadly, and also improving program execution. Examples of barriers in these areas included: lack of monitoring and evaluation tools, structures and processes, and lack of precision planning. These barriers operated at the organizational and individual levels, and the strategies used to address them were found to be effective where applied across contexts. The KIIs illuminated how these challenges were related to information systems and data quality, as well as management and governance issues. Some respondents explained difficulties in even establishing a denominator for campaign and outreach activities to determine coverage indices and assess performance:

*“with the enumeration, we found out that most of the records were not true. The first enumeration we did, someone brought a paper: ‘household 30 children.’ I said ‘haba*!!”- Subnational actor, Nigeria.

Other respondents described gaps in individual and organizational capacity to ensure data quality and effectively utilize available data that needed to be addressed; similarly, at the organizational level respondents described the lack of governance structures to support ongoing performance management and accountability.

Strategies to address these issues included developing mechanisms for feedback, monitoring and evaluation, building robust record systems to capture outcomes, and conducting cyclical small tests of change to refine implementation strategies. On the planning side, working with health staff at the sub-national level to create detailed micro-plans and improve enumeration (sometimes via use of GIS and satellite technology) was universally cited as a key strategy that improved implementation. Micro-plans also put special populations, inaccessible groups and hesitant communities at the center of implementation plans, a tactic which proved critical for ensuring delivery to hard-to-reach populations. Building record systems was an important first step to improving monitoring and evaluation, but those systems proved most functional where ongoing and active engagement with monitoring data occurred. Respondents explained how regular planning sessions became a central program management component. Examples included “situation room” style meetings operationalized via emergency operational centers to examine the level of pre-implementation activities for campaigns, weekly meetings to refine polio surveillance efforts, post-campaign evaluations, and multi-day brainstorming meetings with the objective of creating a roadmap for implementation agreed upon by all stakeholders.

#### Individual, organizational and program characteristics

Individual, organizational and program characteristics accounted for the remainder of barriers to implementation indicated in the survey, as indicated by 18.1%, 11.1% and 9.3% of identified barriers, respectively. These categories covered a broad array of barriers, but included issues related to knowledge and beliefs (individual), poor implementation readiness (organizational), and limited program adaptability (program characteristics). The challenges identified under these categories illustrate how the implementing context and implementation process discussed above are inherently interrelated to the program’s inputs, functions, and actors.

Among respondents who cited individual characteristics as the largest barrier to implementation, in nearly two-thirds of responses (63.3% of barriers related to individual characteristics), an individual’s knowledge and beliefs about the activity (including their attitude toward the polio initiative and their degree of familiarity with facts, truths and principles related to the intervention) was cited as the most significant issue. The KII data suggests this may be a reflection of the social factors that underlie issues related to low acceptability of the polio program and low vaccine uptake. As discussed above, information and communication strategies such as tailored messaging and creating narratives that resonate with community values had to be deployed, in part, at the individual level in order to increase individuals’ knowledge about the polio program, and to resolve issues of distrust and fear held by household decision-makers.

Regarding organizational settings, implementation readiness was the most commonly cited barrier to program implementation (43.9% of barriers related to organizational characteristics). Readiness refers to the availability of resources and capacities, and access to knowledge and information, as well as the level of leadership engagement to ensure the successful delivery of a health program. These challenges occurred at the organizational and policy levels to varying degrees across contexts, depending in large part on the overall strength of the health system. The health system and management gaps discussed above were discussed in detail by KII respondents as indicators of readiness, as were the availability and flow of financial resources. Where resources emanated from was sometimes discussed in relation to the degree of country ownership of the program. One global level respondent explained the potential distorting effect of overreliance on external resources explaining,

*“In the instance of GPEI the money is not going to the government, it’s going to WHO and UNICEF and they are doing jobs that the government should have been doing but they’re basically disincentivizing the government from doing them, surveillance for example, it’s a hard sell to get any government to fund surveillance … we talk about WHO and UNICEF as implementing partners, but they are substituting for government services in that public health system and to get out of that will be very difficult.” –* Global respondent.

Even where program functions were driven by external partners, the level of leadership engagement played a significant role in facilitating program implementation by generating program support and guiding program adaptations to fit the context and health system. From the survey, inability to adapt, tailor or refine the program to meet local needs was the most commonly cited challenge related to the polio program itself (40.2%), and reflects both policy and organizational level constraints.

Where adaptive strategies were successfully applied, they were largely in response to difficulties in reaching hard-to-reach populations (i.e. changing the format, setting or personnel of the intervention), changing epidemiology and vaccine strategy (i.e. adding or removing elements of the program, including the switch from tOPV to bOPV and introduction of IPV), and responding to cultural norms (i.e. adjusting activities, tactics according to local needs). Many adaptive strategies developed over the course of polio eradication had applicability across contexts, for example utilizing environmental surveillance in areas with poor AFP surveillance rates and creating temporary delivery outposts in hard-to-reach areas. By their nature, however, not all strategies developed to address barriers could be universally applied, and indeed, many were specific to the social and political norms of a locality. Door marking, for example, was a monitoring strategy developed by the polio program that was utilized in many contexts to track vaccinations, however who was responsible for marking varied by context, and in the case of Afghanistan, the activity was halted because of objections from local authorities. Reflecting on missed opportunities to apply lessons learned across regions, one respondent in DRC explained:

“*… one of the things that has been criticized is that there was not enough documentation. Obviously from time to time, we talked about it, but it was necessary to document enough so that [others could] appropriate this experience. But locally the actors in the field also developed approaches adapted to their environments … when they had the training, [they could] draw inspiration from others, from what had been done elsewhere, but as far as possible, they took into account local realities.”* – National actor, DRC.

## Discussion

This STRIPE study found that across country contexts and phases, factors external to the polio eradication activities, including political, social, and economic factors, significantly hindered implementation of program activities. Examples of these external factors include lack of social acceptance of polio vaccination due to a confluence of interacting factors at the community level, including mistrust of government and external actors, limited awareness among stakeholders, fatigue with polio-related activities, lack of political will, lack of frameworks conducive to coordinating multi-stakeholder actions, and insecurity and conflict. These external factors played a prominent role because the process of program implementation (planning, executing, engaging, monitoring and evaluating) is largely dependent on them, as well as on the organizational and individual level capacities of various implementers across the socioecological levels. Health systems gaps including human resource shortages, supply chain challenges, and inadequate surveillance systems further hindered the process of program implementation, especially in countries with weak health systems and among hard-to-reach populations.

Over time, the GPEI developed a core set of implementation strategies to address these barriers and facilitate implementation of polio eradication activities, including strategies for social engagement and mobilization, coordinating information and communication at individual and community levels; strategic advocacy at various policy levels; and other health systems strategies such as incentivizing health workers, strengthening data and surveillance systems, micro-planning and establishing emergency operational centers for rapid decision-making. The degree of success of these various strategies depended on the organizational and individual capacities in the different contexts where polio programs were implemented.

While there were positive externalities around the deployment of these strategies (largely around strengthening components of the health system (e.g. supply chain) and building local capacity (e.g. micro-planning), some of these strategies had negative unintended outcomes in certain contexts. These negative unintended outcomes included: distorting the local health systems, especially around the incentive structure of local health workers; inadvertently de-prioritizing other health problems and health system issues in the process of coalescing action and support behind polio eradication goals; creating an unsustainable development of health systems given local resources and priorities, particularly around health system infrastructure and human development; creating an overreliance on external funding and decision-making apparatus at the expense of developing local capacities and resources; and compounding mistrust of government and external actors in some settings.

The STRIPE study findings align very much with the conclusions of other studies or reports that have examined global polio eradication efforts [2–9]. For example, challenges with external factors such as community mistrust leading to vaccine refusals, impact of insecurity and conflict on campaigns and surveillance activities, and the importance of working with community champions have been previously identified by other studies at either a global or national level, or from a specific organizational perspectives [26, 27]. The STRIPE study, however, provides additional insights into these facilitators and barriers by combining experiences at the global, national and sub-national levels and from multiple organizational perspectives. This multi-level and multi-perspective approach allowed for a wholistic view of global polio eradication activities and systematic analyses of how specific challenges emerged or played out across different levels and how strategies were developed in response to those challenges. The STRIPE study particularly prioritized the view of frontline workers at the national and sub-national levels and this yielded important findings that have been less described in the literature, e.g. the significant impact of misconceptions about polio vaccine and the lack of awareness of its benefits among certain frontline workers, their temporary working status, and how these affected specific organizational objectives and the overall polio program goals. The systematic analyses also allowed for a better appreciation of some of the intended and unintended consequences of specific implementation activities within GPEI.

### Lessons learned

Our analyses suggest a few key lessons learned from the polio eradication experience which should inform future global health programs.

First, future eradication or elimination programs must recognize issues related to external factors earlier on, and actively strategize around them on a continuous basis and throughout the life of the program. The extent to which external factors have affected polio eradication activities suggests that global policymakers underestimated the role that differences in political ideology may play in shaping a global health program. It also suggests a limited understanding of the nature of incentives that shape individual, proxy, and collective agency, and reflects an oversimplification of complex and intersecting issues involving politics, culture, and economic disadvantage for different population groups, and how they may change dynamically over time. This lack of understanding and appreciation of the broader political, social and economic contexts surrounding most communities has been and continues to be a major barrier to achieving polio eradication goals.

Second, implementers should conduct careful pre-program analysis of the broader political, social and economic contexts to identify both threats and opportunities to successful program implementation. Had the polio initiative done so, some of the implementation barriers described could have been foreseen and the implementation strategies pre-planned to save time and cost. Instead, many implementation strategies that were deployed under the polio program to address implementation barriers were reactionary as opposed to being preemptive, especially at the national and sub-national levels. Moving forward, readiness assessments of local communities and health systems should predate any global health program such as the GPEI. Such assessments should not only focus on understanding requirements for polio program implementation in a specific context on the supply-side (e.g. health systems inputs such as health workforce, infrastructure, and commodities), but should be extended to understanding social practices, customs, and politics at the local level that could influence the success of the program on the demand-side. Results from these readiness assessments could be used for more effective program planning, to target implementation strategies, and to guide capacity-building activities at various levels, including efforts to build leadership and capacity in management and supervision, to address critical social issues at community levels, and to address other health system gaps.

Third, a set of core principles for implementing complex public health programs emerged across contexts in this study that we feel have relevant application to other health programs. These include: ongoing stakeholder engagement based upon mutual respect, coordinating efforts to build political will and accountability over different phases of the program and at different socioecological levels, systematic adaptation of service delivery activities to local contexts, and establishing sound planning, management and monitoring and evaluation practices which measure implementation outcomes (e.g. acceptability of the various key activities with different stakeholders) in addition to endpoint outcomes (e.g. vaccine coverage under the polio program). It is especially important to identify and work through “gatekeepers” at the community level from the outset of any implementation effort, understand how and when to engage these “gatekeepers,” know the target population and risk factors, and assess ongoing population movement and dynamic changes to the population risk profiles.

Fourth, in order to minimize negative unintended outcomes, policymakers need to give adequate attention to participatory approaches which can facilitate implementation processes across socioecological levels, especially at national and sub-national levels [28, 29]. These approaches could involve participatory planning to align polio eradication goals with other health system objectives at national and sub-national levels and accommodate local knowledge of the context and priorities of community members and frontline health workers; participatory research to understand barriers and identify contextually appropriate solutions to collective goals of the polio program, health system objectives and community priorities; and participatory actions to co-own implementation processes among various stakeholders. If properly managed, these participatory approaches would not only have minimized the negative unintended outcomes of the GPEI, but would also have furthered the positive externalities of the polio eradication goals, contributing to a more efficient and transparent use of resources and improved accountability, as well as helping to address inequities in decision-making processes, resource allocation and utilization, and distribution of the benefits and gains of the polio program over time [28, 29]. Given the multiple contexts involved in the global eradication efforts and the complexities of activities, multiple implementation pathways (i.e. different types and arrangements of implementation activities) for achieving eradication goals should have been anticipated (so as not to assume a “one size fits all” approach to implementation) [30]. In future, these different implementation pathways could be derived from systematic and organized participatory approaches, especially at the national and sub-national levels, which would allow different pathways to be uncovered without compromising coordinated efforts at the global level.

Finally, delivery of lifesaving interventions, no matter how effective, involves individual choices and behaviors, and such choices and behaviors may not always be rational but rather influenced by factors unconnected to disease risks and intervention. This is a reasonable assumption that should underlie any future global disease eradication or control program. Hence, efforts to implement such programs should be accompanied with efforts to build social capital which may address factors outside of the intervention delivery and which may control behaviors and choices at individual and community levels [31].

The lessons learned under global polio eradication efforts described in this paper are some of the first lessons in a series of findings that the STRIPE study aims to contribute to in order to improve the implementation, effectiveness and efficiency of future lifesaving interventions and health system strengthening activities in global health. These lessons are significant for improving health services delivery, overall health outcomes and quality of life of large populations globally. The project also hopes to contribute to the science of implementation and provide real-life data and experiences for formulating theories and frameworks for understanding why interventions may or may not work for their intended purposes, as well as strategies to ensure effective delivery of efficacious interventions under different contexts.

## Conclusion

The implementation of various polio eradication initiatives, including the GPEI, provides important lessons for implementing future lifesaving programs and health systems strengthening activities globally. Understanding the implementing context of any program is critical for identifying both threats and opportunities to program implementation. Systematic efforts to unpack contextual factors, target strategies to perceived barriers, and understand the readiness of recipient communities and health systems should be prioritized before implementation. These systematic efforts are especially relevant at the socioecological levels closest to the point of delivery of the intervention. While polio eradication initiatives were largely successful in deploying various implementation strategies to address barriers to effective implementation of the program, and these strategies yielded positive external benefits, the strategies were only successful where organizational and individual capacity were sufficient, and where they were appropriately tailored to the social, political and administrative context. There were also negative unintended outcomes of the polio eradication initiatives and/or of their related implementation strategies. These negative unintended outcomes could have been minimized with adequate attention to participatory approaches and efforts to build social capital alongside program delivery at national and sub-national levels.

## Supplementary information

**Additional file 1.**

**Additional file 2.**

## Data Availability

Data and publications from this project will be open access and available via an online repository.

## Abbreviations

BMGF: Bill and Melinda Gates foundation
CDC: United States center for disease control
CORE: Collaborations and resources group
DRC: Democratic Republic of Congo
ERIC: Expert recommendation for implementing change framework
GPEI: Global Polio eradication initiative
JHSPH: Johns Hopkins Bloomberg school of public health
KII: Key informant interviews
LMICs: Low- and middle-income countries
NGOs: Non-governmental organization
PAHO: Pan American health organization
STOP: Stop transmission of polio program
STRIPE: Synthesis and translation of research and innovations from polio eradication project
TAC: Technical advisory committee
UNICEF: United Nations international children’s emergency fund
USAID: United States agency for international development
WHO: World health organization

## Footnotes

1 Knowledge assets refers to accumulated knowledge resources, including technical know-how, best practices, ideas, cognitive and technical skills, of individuals within the GPEI and organizations involved in polio eradication broadly.

2 Country epidemiological classifications were determined at the initiation of research, however, as of the time of publication Indonesia and Ethiopia have subsequently confirmed cases of circulating vaccine-derived poliovirus (cVDPV). Indonesia confirmed one case of cVPDV1 in Papua province in November 2018; as of May 2020, Ethiopia had confirmed 22 cases of cVDPV2 linked to an outbreak in the Somali province.
