# Supplementary material for: Synthesis and translation of research and innovations from polio eradication (STRIPE): initial findings from a global mixed methods study
Source: BMC Public Health. 2020 Aug 12;20(Suppl 2):1176. doi: 10.1186/s12889-020-09156-9 (PMC7421832; doi:10.1186/s12889-020-09156-9)
Supplement: Supplementary file 1 — Additional file 1. [file 12889_2020_9156_MOESM1_ESM.docx]

Appendix I

Lessons Learned from Polio

Start of Block: Introduction

Q1.1 Thank you for your participation in the Lessons Learned from the Global Polio Eradication Initiative (GPEI) project. You have been identified as a member of the polio community and we are interested in hearing about your experiences working to eradicate polio. Your insights will be used to inform an understanding of lessons learned from the global initiative to end polio that can be applied to future public health efforts.   **This survey will take about 30 minutes to complete.** Your responses will remain anonymous unless you indicate that you would like to be made available for follow up, including dissemination of survey results and eligibility for an in-depth interview, among other potential activities. Should you include your contact information for follow-up, your personal information and responses will remain confidential and will not be shared beyond the research team without your consent. 
 Are you willing to be reached for follow-up?

- Yes, you may reach me at the following email address: (1) ________________________________________________
- No, I prefer to remain anonymous (2)

End of Block: Introduction

Start of Block: Demographics

Q2.1 Please answer some demographic questions so we can better understand your role within the GPEI.

| 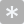 |
| --- |

Q2.2 How long (in years) have you been directly involved with polio eradication activities at any level?

________________________________________________________________

Q2.3 How would you describe the level at which you are/were involved in polio eradication activities? *(Please check all that apply)*

- Global (1)
- National (2)
- State (3)
- District (4)
- Subdistrict (5)

Q2.4 What kind of organization are/were you affiliated with during your involvement in polio eradication activities? *(Please check all that apply)*

- World Health Organization (1)
- Center for Disease Control, US (2)
- Bill and Melinda Gates Foundation (3)
- Rotary International (4)
- UNICEF (5)
- Global NGO (6)
- National implementing partner (7)
- State level implementer (8)
- District level implementer (9)
- Subdistrict level implementer (10)
- National government (11)
- State government (12)
- District government (13)
- Subdistrict government (14)
- Academic/research institution (15)
- Other (please describe) (16) ________________________________________________

Q2.5 Throughout your involvement in polio eradication activities, please **select all countries** that you worked in:

- Global (1)
- WHO Africa Region (2)
- WHO Region of Americas (3)
- WHO South-east Asia Region (4)
- WHO European Region (5)
- WHO Eastern Mediterranean Region (6)
- WHO Western Pacific Region (7)
- Afghanistan (8)
- Albania (9)
- Algeria (10)
- Andorra (11)
- Angola (12)
- Antigua and Barbuda (13)
- Argentina (14)
- Armenia (15)
- Australia (16)
- Austria (17)
- Azerbaijan (18)
- Bahamas (19)
- Bahrain (20)
- Bangladesh (21)
- Barbados (22)
- Belarus (23)
- Belgium (24)
- Belize (25)
- Benin (26)
- Bhutan (27)
- Bolivia (28)
- Bosnia and Herzegovina (29)
- Botswana (30)
- Brazil (31)
- Brunei Darussalam (32)
- Bulgaria (33)
- Burkina Faso (34)
- Burundi (35)
- Cambodia (36)
- Cameroon (37)
- Canada (38)
- Cape Verde (39)
- Central African Republic (40)
- Chad (41)
- Chile (42)
- China (43)
- Colombia (44)
- Comoros (45)
- Congo, Republic of the... (46)
- Costa Rica (47)
- Côte d'Ivoire (48)
- Croatia (49)
- Cuba (50)
- Cyprus (51)
- Czech Republic (52)
- Democratic People's Republic of Korea (53)
- Democratic Republic of the Congo (54)
- Denmark (55)
- Djibouti (56)
- Dominica (57)
- Dominican Republic (58)
- Ecuador (59)
- Egypt (60)
- El Salvador (61)
- Equatorial Guinea (62)
- Eritrea (63)
- Estonia (64)
- Ethiopia (65)
- Fiji (66)
- Finland (67)
- France (68)
- Gabon (69)
- Gambia (70)
- Georgia (71)
- Germany (72)
- Ghana (73)
- Greece (74)
- Grenada (75)
- Guatemala (76)
- Guinea (77)
- Guinea-Bissau (78)
- Guyana (79)
- Haiti (80)
- Honduras (81)
- Hong Kong (S.A.R.) (82)
- Hungary (83)
- Iceland (84)
- India (85)
- Indonesia (86)
- Iran, Islamic Republic of... (87)
- Iraq (88)
- Ireland (89)
- Israel (90)
- Italy (91)
- Jamaica (92)
- Japan (93)
- Jordan (94)
- Kazakhstan (95)
- Kenya (96)
- Kiribati (97)
- Kuwait (98)
- Kyrgyzstan (99)
- Lao People's Democratic Republic (100)
- Latvia (101)
- Lebanon (102)
- Lesotho (103)
- Liberia (104)
- Libyan Arab Jamahiriya (105)
- Liechtenstein (106)
- Lithuania (107)
- Luxembourg (108)
- Madagascar (109)
- Malawi (110)
- Malaysia (111)
- Maldives (112)
- Mali (113)
- Malta (114)
- Marshall Islands (115)
- Mauritania (116)
- Mauritius (117)
- Mexico (118)
- Micronesia, Federated States of... (119)
- Monaco (120)
- Mongolia (121)
- Montenegro (122)
- Morocco (123)
- Mozambique (124)
- Myanmar (125)
- Namibia (126)
- Nauru (127)
- Nepal (128)
- Netherlands (129)
- New Zealand (130)
- Nicaragua (131)
- Niger (132)
- Nigeria (133)
- North Korea (134)
- Norway (135)
- Oman (136)
- Pakistan (137)
- Palau (138)
- Panama (139)
- Papua New Guinea (140)
- Paraguay (141)
- Peru (142)
- Philippines (143)
- Poland (144)
- Portugal (145)
- Qatar (146)
- Republic of Korea (147)
- Republic of Moldova (148)
- Romania (149)
- Russian Federation (150)
- Rwanda (151)
- Saint Kitts and Nevis (152)
- Saint Lucia (153)
- Saint Vincent and the Grenadines (154)
- Samoa (155)
- San Marino (156)
- Sao Tome and Principe (157)
- Saudi Arabia (158)
- Senegal (159)
- Serbia (160)
- Seychelles (161)
- Sierra Leone (162)
- Singapore (163)
- Slovakia (164)
- Slovenia (165)
- Solomon Islands (166)
- Somalia (167)
- South Africa (168)
- South Korea (169)
- Spain (170)
- Sri Lanka (171)
- Sudan (172)
- Suriname (173)
- Swaziland (174)
- Sweden (175)
- Switzerland (176)
- Syrian Arab Republic (177)
- Tajikistan (178)
- Thailand (179)
- The former Yugoslav Republic of Macedonia (180)
- Timor-Leste (181)
- Togo (182)
- Tonga (183)
- Trinidad and Tobago (184)
- Tunisia (185)
- Turkey (186)
- Turkmenistan (187)
- Tuvalu (188)
- Uganda (189)
- Ukraine (190)
- United Arab Emirates (191)
- United Kingdom of Great Britain and Northern Ireland (192)
- United Republic of Tanzania (193)
- United States of America (194)
- Uruguay (195)
- Uzbekistan (196)
- Vanuatu (197)
- Venezuela, Bolivarian Republic of... (198)
- Viet Nam (199)
- Yemen (200)
- Zambia (201)
- Zimbabwe (202)

Q2.6 Which description best defines your role(s) in the implementation of polio eradication activities? (*Please check all that apply)*

- Member at the global level (1)
- Member at a national level (2)
- Member at a subnational level (3)
- Member at the global level (4)
- Member at a national level (5)
- Member at a subnational level (6)
- Program officer (7)
- EPI manager (8)
- Surveillance officer (9)
- Frontline health worker (e.g. vaccinator, community health worker) (10)
- Supervisor (11)
- Program manager (12)
- Country project lead (13)
- Policymaker (14)
- Researcher (15)
- Other (please describe your role) (16) ________________________________________________

Carry Forward Selected Choices from "Which description best defines your role(s) in the implementation of polio eradication activities? (Please check all that apply)"

| 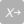 |
| --- |

Q2.7 Of the previously listed roles, which did you hold for the longest period of time? (*Please select the best answer)*

- Member at the global level (1)
- Member at a national level (2)
- Member at a subnational level (3)
- Member at the global level (4)
- Member at a national level (5)
- Member at a subnational level (6)
- Program officer (7)
- EPI manager (8)
- Surveillance officer (9)
- Frontline health worker (e.g. vaccinator, community health worker) (10)
- Supervisor (11)
- Program manager (12)
- Country project lead (13)
- Policymaker (14)
- Researcher (15)
- Other (please describe your role) (16) ________________________________________________

| Page Break |  |
| --- | --- |

Q2.8 During what years did you perform the role of ${Q2.7/ChoiceGroup/SelectedChoices}?

- Start year (1) ________________________________________________
- End year (2) ________________________________________________

Q2.9 In what country/region did you primarily perform the role of ${Q2.7/ChoiceGroup/SelectedChoices}? (*Select the best possible answer)*

- Global (1)
- WHO Africa Region (2)
- WHO Region of Americas (3)
- WHO South-east Asia Region (4)
- WHO European Region (5)
- WHO Eastern Mediterranean Region (6)
- WHO Western Pacific Region (7)
- Afghanistan (8)
- Albania (9)
- Algeria (10)
- Andorra (11)
- Angola (12)
- Antigua and Barbuda (13)
- Argentina (14)
- Armenia (15)
- Australia (16)
- Austria (17)
- Azerbaijan (18)
- Bahamas (19)
- Bahrain (20)
- Bangladesh (21)
- Barbados (22)
- Belarus (23)
- Belgium (24)
- Belize (25)
- Benin (26)
- Bhutan (27)
- Bolivia (28)
- Bosnia and Herzegovina (29)
- Botswana (30)
- Brazil (31)
- Brunei Darussalam (32)
- Bulgaria (33)
- Burkina Faso (34)
- Burundi (35)
- Cambodia (36)
- Cameroon (37)
- Canada (38)
- Cape Verde (39)
- Central African Republic (40)
- Chad (41)
- Chile (42)
- China (43)
- Colombia (44)
- Comoros (45)
- Congo, Republic of the... (46)
- Costa Rica (47)
- Côte d'Ivoire (48)
- Croatia (49)
- Cuba (50)
- Cyprus (51)
- Czech Republic (52)
- Democratic People's Republic of Korea (53)
- Democratic Republic of the Congo (54)
- Denmark (55)
- Djibouti (56)
- Dominica (57)
- Dominican Republic (58)
- Ecuador (59)
- Egypt (60)
- El Salvador (61)
- Equatorial Guinea (62)
- Eritrea (63)
- Estonia (64)
- Ethiopia (65)
- Fiji (66)
- Finland (67)
- France (68)
- Gabon (69)
- Gambia (70)
- Georgia (71)
- Germany (72)
- Ghana (73)
- Greece (74)
- Grenada (75)
- Guatemala (76)
- Guinea (77)
- Guinea-Bissau (78)
- Guyana (79)
- Haiti (80)
- Honduras (81)
- Hong Kong (S.A.R.) (82)
- Hungary (83)
- Iceland (84)
- India (85)
- Indonesia (86)
- Iran, Islamic Republic of... (87)
- Iraq (88)
- Ireland (89)
- Israel (90)
- Italy (91)
- Jamaica (92)
- Japan (93)
- Jordan (94)
- Kazakhstan (95)
- Kenya (96)
- Kiribati (97)
- Kuwait (98)
- Kyrgyzstan (99)
- Lao People's Democratic Republic (100)
- Latvia (101)
- Lebanon (102)
- Lesotho (103)
- Liberia (104)
- Libyan Arab Jamahiriya (105)
- Liechtenstein (106)
- Lithuania (107)
- Luxembourg (108)
- Madagascar (109)
- Malawi (110)
- Malaysia (111)
- Maldives (112)
- Mali (113)
- Malta (114)
- Marshall Islands (115)
- Mauritania (116)
- Mauritius (117)
- Mexico (118)
- Micronesia, Federated States of... (119)
- Monaco (120)
- Mongolia (121)
- Montenegro (122)
- Morocco (123)
- Mozambique (124)
- Myanmar (125)
- Namibia (126)
- Nauru (127)
- Nepal (128)
- Netherlands (129)
- New Zealand (130)
- Nicaragua (131)
- Niger (132)
- Nigeria (133)
- North Korea (134)
- Norway (135)
- Oman (136)
- Pakistan (137)
- Palau (138)
- Panama (139)
- Papua New Guinea (140)
- Paraguay (141)
- Peru (142)
- Philippines (143)
- Poland (144)
- Portugal (145)
- Qatar (146)
- Republic of Korea (147)
- Republic of Moldova (148)
- Romania (149)
- Russian Federation (150)
- Rwanda (151)
- Saint Kitts and Nevis (152)
- Saint Lucia (153)
- Saint Vincent and the Grenadines (154)
- Samoa (155)
- San Marino (156)
- Sao Tome and Principe (157)
- Saudi Arabia (158)
- Senegal (159)
- Serbia (160)
- Seychelles (161)
- Sierra Leone (162)
- Singapore (163)
- Slovakia (164)
- Slovenia (165)
- Solomon Islands (166)
- Somalia (167)
- South Africa (168)
- South Korea (169)
- Spain (170)
- Sri Lanka (171)
- Sudan (172)
- Suriname (173)
- Swaziland (174)
- Sweden (175)
- Switzerland (176)
- Syrian Arab Republic (177)
- Tajikistan (178)
- Thailand (179)
- The former Yugoslav Republic of Macedonia (180)
- Timor-Leste (181)
- Togo (182)
- Tonga (183)
- Trinidad and Tobago (184)
- Tunisia (185)
- Turkey (186)
- Turkmenistan (187)
- Tuvalu (188)
- Uganda (189)
- Ukraine (190)
- United Arab Emirates (191)
- United Kingdom of Great Britain and Northern Ireland (192)
- United Republic of Tanzania (193)
- United States of America (194)
- Uruguay (195)
- Uzbekistan (196)
- Vanuatu (197)
- Venezuela, Bolivarian Republic of... (198)
- Viet Nam (199)
- Yemen (200)
- Zambia (201)
- Zimbabwe (202)

End of Block: Demographics

Start of Block: Primary Role

Q3.1 For the following sections, please describe your experiences with GPEI activities in your role as ${Q2.7/ChoiceGroup/SelectedChoices} from ${Q2.8/ChoiceGroup/AllChoicesTextEntry} in ${Q2.9/ChoiceGroup/SelectedChoices}

| 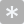 |
| --- |

Q3.2 What was/were the objective(s) of the polio eradication activities you were involved in for that role, in that country? (*Please check up to three objectives)*

- Resource mobilization: Goal to mobilize sufficient funds at global, national and/or subnational levels to complete GPEI-related activities. (1)
- Partnership/alliance development: Goal to develop relationships with and/or among stakeholders, including donors, implementing organizations, private sector organizations, civil society, government ministries and representative bodies. (2)
- Strategy development and planning: Efforts to identify appropriate implementation strategies, develop implementation plans for national and subnational contexts. (3)
- Strengthening delivery systems: Goal to develop delivery systems to enable vaccination at the right time, for the right populations. Efforts may include developing infrastructure; recruiting, training, and supervising personnel; strengthening supply chains, etc. (4)
- Vaccination: Goal to vaccinate populations with poliovirus vaccines, either through routine immunizations, supplementary immunization activities, or targeted mop-up campaigns. (5)
- Surveillance: Goal to conduct AFP and environmental surveillance, conduct case report follow-up, and strengthen surveillance network capacities. Efforts may include seroprevalence studies, environmental sampling, stool sample transport and testing, lab capacity development, etc. (6)
- Community engagement: Goal to improve demand via improved outreach, behavioral change interventions, collaboration with community leaders, efforts to counteract misinformation and mistrust. (7)
- Monitoring & Evaluating: Goal to monitor program activities, progress and evaluate program fidelity, outcomes and impact (8)
- Other: (please describe briefly) (9) ________________________________________________

End of Block: Primary Role

Start of Block: Resource Mobilization

Q4.1 *For this next set of questions, we would like to ask you about implementation facilitators and challenges relevant to the objective of* ***Resource Mobilization***

Q4.2 In your opinion, which of the following was the biggest ***internal*** ***contributor*** to your program's success in completing its objective of Resource Mobilization*?*

- Characteristics of individuals within your organization involved in polio eradication activities (1)
- Organizational settings (factors related to your organization supporting the polio eradication program) (2)
- Polio eradication program characteristics (the activity that was used towards eradicating polio, including technologies that were adopted by the organization/individual implementing the activities) (3)
- Process of conducting the activities (how the activity was implemented, including the planning, execution strategies, reflection and evaluation of activities, or adjustments made to the plan) (4)

Q4.3 In your opinion, which of the following was the biggest ***external contributor*** to your program’s success in completing its objective of Resource Mobilization*?*

- Political environment (lawmaker support, political climate accepting of polio eradication activities, and political structure to conducive to coordinated action) (1)
- Economic environment (sufficient revenue sources/base to fund activities and/or maintain system developments) (2)
- Social environment (social norms around immunization, accepting communities in which polio eradication activities were implemented) (3)
- Technological environment (infrastructure or technological advances outside of the organization) (4)
- Other environment (Please describe): (5) ________________________________________________

Q4.4 Please briefly describe the most influential internal, external or combination of contributors to your program's success in completing its objective of resource mobilization

________________________________________________________________

________________________________________________________________

________________________________________________________________

________________________________________________________________

________________________________________________________________

Q4.5 Which of the following best describes where you experienced significant ***challenges*** when carrying out activities associated with Resource Mobilization? (*Please* *check all that apply)*

- Characteristics of individuals associated with your organization involved in polio eradication activities (1)
- Organizational settings (factors related to your organization supporting the polio eradication program) (2)
- Polio eradication program characteristics (the activity that was used towards eradicating polio) (3)
- Process of conducting the activities (how the activity was implemented, including the planning, execution strategies, reflection and evaluation of activities, or adjustments made to the plan) (4)
- External settings (political, economic, social, technological or environmental settings) (5)

Display This Question:

If Which of the following best describes where you experienced significant challenges when carrying... = Characteristics of individuals associated with your organization involved in polio eradication activities

Q4.6 What characteristic(s) best describes implementation challenges to Resource Mobilization you encountered related to an **individual’s characteristics**? (*Please* *check all that apply)*

- The person’s knowledge and beliefs about the activity (individuals did not have a positive attitude towards the polio eradication activity, were unfamiliar with facts, truths and principles related to the intervention) (1)
- Self-efficacy (individuals did not have belief in own abilities to execute courses of action to achieve polio eradication goals) (2)
- Individual stage of change (how likely the individual is towards skilled, enthusiastic and sustained support of the polio eradication activities.) (3)
- Individual identification with organization (poor perception of the organization and poor relationship/commitment to the organization) (4)
- Other personal attributes (please describe): (5) ________________________________________________

Display This Question:

If Which of the following best describes where you experienced significant challenges when carrying... = Organizational settings (factors related to your organization supporting the polio eradication program)

Q4.7 What characteristic(s) best describes implementation challenges to Resource Mobilization you encountered related to your **organizational setting**? (*Please* *check all that apply)*

- Structural characteristics (age, social architecture, size of an organization led to challenges) (1)
- Networks and communications (nature and quality of formal and informal communication in an organization led to challenges) (2)
- Culture (norms, values, basic assumptions of an organization led to challenges) (3)
- Implementation climate (capacity for change, receptivity of team to proposed intervention, relative priority of project, organizational goals, incentive and rewards, etc. led to challenges) (4)
- Readiness for implementation (Level of leadership engagement, available resources, access to knowledge and information led to challenges) (5)
- Other personal attributes (please describe): (6) ________________________________________________

Display This Question:

If Which of the following best describes where you experienced significant challenges when carrying... = Polio eradication program characteristics (the activity that was used towards eradicating polio)

Q4.8 What characteristic(s) best capture implementation challenges to Resource Mobilization you encountered related to **GPEI program characteristics**? (*Please* *check all that apply)*

- Intervention source (perception that intervention developed either internally or externally led to challenges) (1)
- Evidence strength and quality (perceptions of the quality and validity of evidence did not support belief that the intervention will have desired outcomes) (2)
- Relative advantage (perceptions that there was another, better approach) (3)
- Adaptability to local context (The activity was not adapted, tailored or refined to meet local needs) (4)
- Trialability (no ability to test on a small scale and reverse course if warranted) (5)
- Complexity (perceived difficulty of implementation reflected by duration, scope, radicalness, disruptiveness, centrality, and intricacy and number of steps required to implement) (6)
- Design Quality and Packaging (difficulty arising from how the intervention is bundled, presented, and assembled) (7)
- Cost (including cost of intervention, cost of implementing intervention, including investment, supply, and opportunity costs) (8)
- Other (please describe): (9) ________________________________________________

Display This Question:

If Which of the following best describes where you experienced significant challenges when carrying... = Process of conducting the activities (how the activity was implemented, including the planning, execution strategies, reflection and evaluation of activities, or adjustments made to the plan)

Q4.9 What stage(s) of the **process of program implementation** did you experience implementation challenges to Resource Mobilization? (*Please* *check all that apply)*

- Planning (degree to which schemes/methods of implementing activities are developed in advance or poor quality of such methods) (1)
- Engaging (attracting and involving appropriate individuals or organizations in implementation of polio eradication activities) (2)
- Executing (carrying out activities according to plan) (3)
- Reflecting and evaluating (monitoring and feedback about the progress and quality of implementation accompanied with regular personal and team debriefing about progress and experience) (4)
- Other (please describe): (5) ________________________________________________

Display This Question:

If Which of the following best describes where you experienced significant challenges when carrying... = External settings (political, economic, social, technological or environmental settings)

Q4.10 What characteristic(s) best capture implementation challenges of Resource Mobilization you encountered related to **external settings**? (*Please* *check all that apply)*

- Political environment (Policymaker disinterest or resistance, limited windows of opportunity within the political climate, political structure non-conducive to coordinated action.) (1)
- Economic environment (Insufficient revenue sources/base to fund activities and/or maintain system developments) (2)
- Social environment (Communities in which polio eradication activities were implemented are non-accepting and/or resistant to intervention) (3)
- Technological environment (Slow or limited advances of technologies used in implementing polio eradication activities) (4)
- Other environment (environment where activity was implemented was prohibitive and did not contribute to the success of polio eradication, including the global climate and ineffective cross-organizational collaboration) (5)

Q4.11 Please describe the most influential internal, external or combination of challenges you experienced when carrying out activities associated with resource mobilization

________________________________________________________________

________________________________________________________________

________________________________________________________________

________________________________________________________________

________________________________________________________________

End of Block: Resource Mobilization

Start of Block: Partnership/Alliance Development

Q5.1 *For this next set of questions, we would like to ask you about implementation facilitators and challenges relevant to the objective of* ***Partnership/Alliance Development***

Q5.2 In your opinion, which of the following was the biggest ***internal*** ***contributor*** to your program's success in completing its objective of Partnership/Alliance development*?*

- Characteristics of individuals within your organization involved in polio eradication activities (1)
- Organizational settings (factors related to your organization supporting the polio eradication program) (2)
- Polio eradication program characteristics (the activity that was used towards eradicating polio, including technologies that were adopted by the organization/individual implementing the activities) (3)
- Process of conducting the activities (how the activity was implemented, including the planning, execution strategies, reflection and evaluation of activities, or adjustments made to the plan) (4)

Q5.3 In your opinion, which of the following was the biggest ***external contributor*** to your program’s success in completing its objective of Partnership/Alliance development*?*

- Political environment (lawmaker support, political climate accepting of polio eradication activities, and political structure to conducive to coordinated action) (1)
- Economic environment (sufficient revenue sources/base to fund activities and/or maintain system developments) (2)
- Social environment (social norms around immunization, accepting communities in which polio eradication activities were implemented) (3)
- Technological environment (infrastructure or technological advances outside of the organization) (4)
- Other environment (Please describe): (5) ________________________________________________

Q5.4 Please briefly describe the most influential internal, external or combination of contributors to your program's success in completing its objective of Partnership/Alliance development

________________________________________________________________

________________________________________________________________

________________________________________________________________

________________________________________________________________

________________________________________________________________

Q5.5 Which of the following best describes where you experienced significant ***challenges*** when carrying out activities associated with Partnership/Alliance development? (*Please* *check all that apply)*

- Characteristics of individuals associated with your organization involved in polio eradication activities (1)
- Organizational settings (factors related to your organization supporting the polio eradication program) (2)
- Polio eradication program characteristics (the activity that was used towards eradicating polio) (3)
- Process of conducting the activities (how the activity was implemented, including the planning, execution strategies, reflection and evaluation of activities, or adjustments made to the plan) (4)
- External settings (political, economic, social, technological or environmental settings) (5)

Display This Question:

If Which of the following best describes where you experienced significant challenges when carrying... = Characteristics of individuals associated with your organization involved in polio eradication activities

Q5.6 What characteristic(s) best describes implementation challenges of Partnership/Alliance development you encountered related to an **individual’s characteristics**? (*Please* *check all that apply)*

- The person’s knowledge and beliefs about the activity (individuals did not have a positive attitude towards the polio eradication activity, were unfamiliar with facts, truths and principles related to the intervention) (1)
- Self-efficacy (individuals did not have belief in own abilities to execute courses of action to achieve polio eradication goals) (2)
- Individual stage of change (how likely the individual is towards skilled, enthusiastic and sustained support of the polio eradication activities.) (3)
- Individual identification with organization (poor perception of the organization and poor relationship/commitment to the organization) (4)
- Other personal attributes (please describe): (5) ________________________________________________

Display This Question:

If Which of the following best describes where you experienced significant challenges when carrying... = Organizational settings (factors related to your organization supporting the polio eradication program)

Q5.7 What characteristic(s) best describes implementation challenges of Partnership/Alliance development you encountered related to your **organizational setting**? (*Please* *check all that apply)*

- Structural characteristics (age, social architecture, size of an organization led to challenges) (1)
- Networks and communications (nature and quality of formal and informal communication in an organization led to challenges) (2)
- Culture (norms, values, basic assumptions of an organization led to challenges) (3)
- Implementation climate (capacity for change, receptivity of team to proposed intervention, relative priority of project, organizational goals, incentive and rewards, etc. led to challenges) (4)
- Readiness for implementation (Level of leadership engagement, available resources, access to knowledge and information led to challenges) (5)
- Other personal attributes (please describe): (6) ________________________________________________

Display This Question:

If Which of the following best describes where you experienced significant challenges when carrying... = Polio eradication program characteristics (the activity that was used towards eradicating polio)

Q5.8 What characteristic(s) best capture implementation challenges of Partnership/Alliance development you encountered related to **GPEI program characteristics**? (*Please* *check all that apply)*

- Intervention source (perception that intervention developed either internally or externally led to challenges) (1)
- Evidence strength and quality (perceptions of the quality and validity of evidence did not support belief that the intervention will have desired outcomes) (2)
- Relative advantage (perceptions that there was another, better approach) (3)
- Adaptability to local context (The activity was not adapted, tailored or refined to meet local needs) (4)
- Trialability (no ability to test on a small scale and reverse course if warranted) (5)
- Complexity (perceived difficulty of implementation reflected by duration, scope, radicalness, disruptiveness, centrality, and intricacy and number of steps required to implement) (6)
- Design Quality and Packaging (difficulty arising from how the intervention is bundled, presented, and assembled) (7)
- Cost (including cost of intervention, cost of implementing intervention, including investment, supply, and opportunity costs) (8)
- Other (please describe): (9) ________________________________________________

Display This Question:

If Which of the following best describes where you experienced significant challenges when carrying... = Process of conducting the activities (how the activity was implemented, including the planning, execution strategies, reflection and evaluation of activities, or adjustments made to the plan)

Q5.9 What stage(s) of the **process of program implementation** did you experience implementation challenges of Partnership/Alliance development? (*Please* *check all that apply)*

- Planning (degree to which schemes/methods of implementing activities are developed in advance or poor quality of such methods) (1)
- Engaging (attracting and involving appropriate individuals or organizations in implementation of polio eradication activities) (2)
- Executing (carrying out activities according to plan) (3)
- Reflecting and evaluating (monitoring and feedback about the progress and quality of implementation accompanied with regular personal and team debriefing about progress and experience) (4)
- Other (please describe): (5) ________________________________________________

Display This Question:

If Which of the following best describes where you experienced significant challenges when carrying... = External settings (political, economic, social, technological or environmental settings)

Q5.10 What characteristic(s) best capture implementation challenges of Partnership/Alliance development you encountered related to **external settings**? (*Please* *check all that apply)*

- Political environment (Policymaker disinterest or resistance, limited windows of opportunity within the political climate, political structure non-conducive to coordinated action.) (1)
- Economic environment (Insufficient revenue sources/base to fund activities and/or maintain system developments) (2)
- Social environment (Communities in which polio eradication activities were implemented are non-accepting and/or resistant to intervention) (3)
- Technological environment (Slow or limited advances of technologies used in implementing polio eradication activities) (4)
- Other environment (environment where activity was implemented was prohibitive and did not contribute to the success of polio eradication, including the global climate and ineffective cross-organizational collaboration) (5)

Q5.11 Please describe the most influential internal, external or combination of challenges you experienced when carrying out activities associated with partnership/alliance development

________________________________________________________________

________________________________________________________________

________________________________________________________________

________________________________________________________________

________________________________________________________________

End of Block: Partnership/Alliance Development

Start of Block: Strategy Development and Planning

Q6.1 *For this next set of questions, we would like to ask you about implementation facilitators and challenges relevant to the objective of* ***Strategy Development and Planning***

Q6.2 In your opinion, which of the following was the biggest ***internal*** ***contributor*** to your program's success in completing its objective of Strategy Development and Planning*?*

- Characteristics of individuals within your organization involved in polio eradication activities (1)
- Organizational settings (factors related to your organization supporting the polio eradication program) (2)
- Polio eradication program characteristics (the activity that was used towards eradicating polio, including technologies that were adopted by the organization/individual implementing the activities) (3)
- Process of conducting the activities (how the activity was implemented, including the planning, execution strategies, reflection and evaluation of activities, or adjustments made to the plan) (4)

Q6.3 In your opinion, which of the following was the biggest ***external contributor*** to your program’s success in completing its objective of Strategy Development and Planning*?*

- Political environment (lawmaker support, political climate accepting of polio eradication activities, and political structure to conducive to coordinated action) (1)
- Economic environment (sufficient revenue sources/base to fund activities and/or maintain system developments) (2)
- Social environment (social norms around immunization, accepting communities in which polio eradication activities were implemented) (3)
- Technological environment (infrastructure or technological advances outside of the organization) (4)
- Other environment (Please describe): (5) ________________________________________________

Q6.4 Please briefly describe the most influential internal, external or combination of contributors to your program's success in completing its objective of Strategy Development and Planning

________________________________________________________________

________________________________________________________________

________________________________________________________________

________________________________________________________________

________________________________________________________________

Q6.5 Which of the following best describes where you experienced significant ***challenges*** when carrying out activities associated with Strategy Development and Planning? (*Please* *check all that apply)*

- Characteristics of individuals associated with your organization involved in polio eradication activities (1)
- Organizational settings (factors related to your organization supporting the polio eradication program) (2)
- Polio eradication program characteristics (the activity that was used towards eradicating polio) (3)
- Process of conducting the activities (how the activity was implemented, including the planning, execution strategies, reflection and evaluation of activities, or adjustments made to the plan) (4)
- External settings (political, economic, social, technological or environmental settings) (5)

Display This Question:

If Which of the following best describes where you experienced significant challenges when carrying... = Characteristics of individuals associated with your organization involved in polio eradication activities

Q6.6 What characteristic(s) best describes implementation challenges to Strategy Development and Planning you encountered related to an **individual’s characteristics**? (*Please* *check all that apply)*

- The person’s knowledge and beliefs about the activity (individuals did not have a positive attitude towards the polio eradication activity, were unfamiliar with facts, truths and principles related to the intervention) (1)
- Self-efficacy (individuals did not have belief in own abilities to execute courses of action to achieve polio eradication goals) (2)
- Individual stage of change (how likely the individual is towards skilled, enthusiastic and sustained support of the polio eradication activities.) (3)
- Individual identification with organization (poor perception of the organization and poor relationship/commitment to the organization) (4)
- Other personal attributes (please describe): (5) ________________________________________________

Display This Question:

If Which of the following best describes where you experienced significant challenges when carrying... = Organizational settings (factors related to your organization supporting the polio eradication program)

Q6.7 What characteristic(s) best describes implementation challenges to Strategy Development and Planning you encountered related to your **organizational setting**? (*Please* *check all that apply)*

- Structural characteristics (age, social architecture, size of an organization led to challenges) (1)
- Networks and communications (nature and quality of formal and informal communication in an organization led to challenges) (2)
- Culture (norms, values, basic assumptions of an organization led to challenges) (3)
- Implementation climate (capacity for change, receptivity of team to proposed intervention, relative priority of project, organizational goals, incentive and rewards, etc. led to challenges) (4)
- Readiness for implementation (Level of leadership engagement, available resources, access to knowledge and information led to challenges) (5)
- Other personal attributes (please describe): (6) ________________________________________________

Display This Question:

If Which of the following best describes where you experienced significant challenges when carrying... = Polio eradication program characteristics (the activity that was used towards eradicating polio)

Q6.8 What characteristic(s) best capture implementation challenges to Strategy Development and Planning you encountered related to **GPEI program characteristics**? (*Please* *check all that apply)*

- Intervention source (perception that intervention developed either internally or externally led to challenges) (1)
- Evidence strength and quality (perceptions of the quality and validity of evidence did not support belief that the intervention will have desired outcomes) (2)
- Relative advantage (perceptions that there was another, better approach) (3)
- Adaptability to local context (The activity was not adapted, tailored or refined to meet local needs) (4)
- Trialability (no ability to test on a small scale and reverse course if warranted) (5)
- Complexity (perceived difficulty of implementation reflected by duration, scope, radicalness, disruptiveness, centrality, and intricacy and number of steps required to implement) (6)
- Design Quality and Packaging (difficulty arising from how the intervention is bundled, presented, and assembled) (7)
- Cost (including cost of intervention, cost of implementing intervention, including investment, supply, and opportunity costs) (8)
- Other (please describe): (9) ________________________________________________

Display This Question:

If Which of the following best describes where you experienced significant challenges when carrying... = Process of conducting the activities (how the activity was implemented, including the planning, execution strategies, reflection and evaluation of activities, or adjustments made to the plan)

Q6.9 What stage(s) of the **process of program implementation** did you experience implementation challenges to Strategy Development and Planning? (*Please* *check all that apply)*

- Planning (degree to which schemes/methods of implementing activities are developed in advance or poor quality of such methods) (1)
- Engaging (attracting and involving appropriate individuals or organizations in implementation of polio eradication activities) (2)
- Executing (carrying out activities according to plan) (3)
- Reflecting and evaluating (monitoring and feedback about the progress and quality of implementation accompanied with regular personal and team debriefing about progress and experience) (4)
- Other (please describe): (5) ________________________________________________

Display This Question:

If Which of the following best describes where you experienced significant challenges when carrying... = External settings (political, economic, social, technological or environmental settings)

Q6.10 What characteristic(s) best capture implementation challenges of Strategy Development and Planning you encountered related to **external settings**? (*Please* *check all that apply)*

- Political environment (Policymaker disinterest or resistance, limited windows of opportunity within the political climate, political structure non-conducive to coordinated action.) (1)
- Economic environment (Insufficient revenue sources/base to fund activities and/or maintain system developments) (2)
- Social environment (Communities in which polio eradication activities were implemented are non-accepting and/or resistant to intervention) (3)
- Technological environment (Slow or limited advances of technologies used in implementing polio eradication activities) (4)
- Other environment (environment where activity was implemented was prohibitive and did not contribute to the success of polio eradication, including the global climate and ineffective cross-organizational collaboration) (5)

Q6.11 Please describe the most influential internal, external or combination of challenges you experienced when carrying out activities associated with Strategy Development and Planning

________________________________________________________________

________________________________________________________________

________________________________________________________________

________________________________________________________________

________________________________________________________________

End of Block: Strategy Development and Planning

Start of Block: Strengthening Delivery Systems

Q7.1 *For this next set of questions, we would like to ask you about implementation facilitators and challenges relevant to the objective of***Strengthening Delivery Systems**

Q7.2 In your opinion, which of the following was the biggest ***internal*** ***contributor*** to your program's success in completing its objective of Strengthening Delivery Systems*?*

- Characteristics of individuals within your organization involved in polio eradication activities (1)
- Organizational settings (factors related to your organization supporting the polio eradication program) (2)
- Polio eradication program characteristics (the activity that was used towards eradicating polio, including technologies that were adopted by the organization/individual implementing the activities) (3)
- Process of conducting the activities (how the activity was implemented, including the planning, execution strategies, reflection and evaluation of activities, or adjustments made to the plan) (4)

Q7.3 In your opinion, which of the following was the biggest ***external contributor*** to your program’s success in completing its objective of Strengthening Delivery Systems*?*

- Political environment (lawmaker support, political climate accepting of polio eradication activities, and political structure to conducive to coordinated action) (1)
- Economic environment (sufficient revenue sources/base to fund activities and/or maintain system developments) (2)
- Social environment (social norms around immunization, accepting communities in which polio eradication activities were implemented) (3)
- Technological environment (infrastructure or technological advances outside of the organization) (4)
- Other environment (Please describe): (5) ________________________________________________

Q7.4 Please briefly describe the most influential internal, external or combination of contributors to your program's success in completing its objective of Strengthening Delivery Systems

________________________________________________________________

________________________________________________________________

________________________________________________________________

________________________________________________________________

________________________________________________________________

Q7.5 Which of the following best describes where you experienced significant ***challenges*** when carrying out activities associated with Strengthening Delivery Systems? (*Please* *check all that apply)*

- Characteristics of individuals associated with your organization involved in polio eradication activities (1)
- Organizational settings (factors related to your organization supporting the polio eradication program) (2)
- Polio eradication program characteristics (the activity that was used towards eradicating polio) (3)
- Process of conducting the activities (how the activity was implemented, including the planning, execution strategies, reflection and evaluation of activities, or adjustments made to the plan) (4)
- External settings (political, economic, social, technological or environmental settings) (5)

Display This Question:

If Which of the following best describes where you experienced significant challenges when carrying... = Characteristics of individuals associated with your organization involved in polio eradication activities

Q7.6 What characteristic(s) best describes implementation challenges to Strengthening Delivery Systems you encountered related to an **individual’s characteristics**? (*Please* *check all that apply)*

- The person’s knowledge and beliefs about the activity (individuals did not have a positive attitude towards the polio eradication activity, were unfamiliar with facts, truths and principles related to the intervention) (1)
- Self-efficacy (individuals did not have belief in own abilities to execute courses of action to achieve polio eradication goals) (2)
- Individual stage of change (how likely the individual is towards skilled, enthusiastic and sustained support of the polio eradication activities.) (3)
- Individual identification with organization (poor perception of the organization and poor relationship/commitment to the organization) (4)
- Other personal attributes (please describe): (5) ________________________________________________

Display This Question:

If Which of the following best describes where you experienced significant challenges when carrying... = Organizational settings (factors related to your organization supporting the polio eradication program)

Q7.7 What characteristic(s) best describes implementation challenges to Strengthening Delivery Systems you encountered related to your **organizational setting**? (*Please* *check all that apply)*

- Structural characteristics (age, social architecture, size of an organization led to challenges) (1)
- Networks and communications (nature and quality of formal and informal communication in an organization led to challenges) (2)
- Culture (norms, values, basic assumptions of an organization led to challenges) (3)
- Implementation climate (capacity for change, receptivity of team to proposed intervention, relative priority of project, organizational goals, incentive and rewards, etc. led to challenges) (4)
- Readiness for implementation (Level of leadership engagement, available resources, access to knowledge and information led to challenges) (5)
- Other personal attributes (please describe): (6) ________________________________________________

Display This Question:

If Which of the following best describes where you experienced significant challenges when carrying... = Polio eradication program characteristics (the activity that was used towards eradicating polio)

Q7.8 What characteristic(s) best capture implementation challenges to Strengthening Delivery Systems you encountered related to **GPEI program characteristics**? (*Please* *check all that apply)*

- Intervention source (perception that intervention developed either internally or externally led to challenges) (1)
- Evidence strength and quality (perceptions of the quality and validity of evidence did not support belief that the intervention will have desired outcomes) (2)
- Relative advantage (perceptions that there was another, better approach) (3)
- Adaptability to local context (The activity was not adapted, tailored or refined to meet local needs) (4)
- Trialability (no ability to test on a small scale and reverse course if warranted) (5)
- Complexity (perceived difficulty of implementation reflected by duration, scope, radicalness, disruptiveness, centrality, and intricacy and number of steps required to implement) (6)
- Design Quality and Packaging (difficulty arising from how the intervention is bundled, presented, and assembled) (7)
- Cost (including cost of intervention, cost of implementing intervention, including investment, supply, and opportunity costs) (8)
- Other (please describe): (9) ________________________________________________

Display This Question:

If Which of the following best describes where you experienced significant challenges when carrying... = Process of conducting the activities (how the activity was implemented, including the planning, execution strategies, reflection and evaluation of activities, or adjustments made to the plan)

Q7.9 What stage(s) of the **process of program implementation** did you experience implementation challenges to Strengthening Delivery Systems? (*Please* *check all that apply)*

- Planning (degree to which schemes/methods of implementing activities are developed in advance or poor quality of such methods) (1)
- Engaging (attracting and involving appropriate individuals or organizations in implementation of polio eradication activities) (2)
- Executing (carrying out activities according to plan) (3)
- Reflecting and evaluating (monitoring and feedback about the progress and quality of implementation accompanied with regular personal and team debriefing about progress and experience) (4)
- Other (please describe): (5) ________________________________________________

Display This Question:

If Which of the following best describes where you experienced significant challenges when carrying... = External settings (political, economic, social, technological or environmental settings)

Q7.10 What characteristic(s) best capture implementation challenges of Strengthening Delivery Systems you encountered related to **external settings**? (*Please* *check all that apply)*

- Political environment (Policymaker disinterest or resistance, limited windows of opportunity within the political climate, political structure non-conducive to coordinated action.) (1)
- Economic environment (Insufficient revenue sources/base to fund activities and/or maintain system developments) (2)
- Social environment (Communities in which polio eradication activities were implemented are non-accepting and/or resistant to intervention) (3)
- Technological environment (Slow or limited advances of technologies used in implementing polio eradication activities) (4)
- Other environment (environment where activity was implemented was prohibitive and did not contribute to the success of polio eradication, including the global climate and ineffective cross-organizational collaboration) (5)

Q7.11 Please describe the most influential internal, external or combination of challenges you experienced when carrying out activities associated with Strengthening Delivery Systems

________________________________________________________________

________________________________________________________________

________________________________________________________________

________________________________________________________________

________________________________________________________________

End of Block: Strengthening Delivery Systems

Start of Block: Vaccination

Q8.1 *For this next set of questions, we would like to ask you about implementation facilitators and challenges relevant to the objective of* ***Vaccination***

Q8.2 In your opinion, which of the following was the biggest ***internal*** ***contributor*** to your program's success in completing its objective of Vaccination*?*

- Characteristics of individuals within your organization involved in polio eradication activities (1)
- Organizational settings (factors related to your organization supporting the polio eradication program) (2)
- Polio eradication program characteristics (the activity that was used towards eradicating polio, including technologies that were adopted by the organization/individual implementing the activities) (3)
- Process of conducting the activities (how the activity was implemented, including the planning, execution strategies, reflection and evaluation of activities, or adjustments made to the plan) (4)

Q8.3 In your opinion, which of the following was the biggest ***external contributor*** to your program’s success in completing its objective of Vaccination*?*

- Political environment (lawmaker support, political climate accepting of polio eradication activities, and political structure to conducive to coordinated action) (1)
- Economic environment (sufficient revenue sources/base to fund activities and/or maintain system developments) (2)
- Social environment (social norms around immunization, accepting communities in which polio eradication activities were implemented) (3)
- Technological environment (infrastructure or technological advances outside of the organization) (4)
- Other environment (Please describe): (5) ________________________________________________

Q8.4 Please briefly describe the most influential internal, external or combination of contributors to your program's success in completing its objective of Vaccination

________________________________________________________________

________________________________________________________________

________________________________________________________________

________________________________________________________________

________________________________________________________________

Q8.5 Which of the following best describes where you experienced significant ***challenges*** when carrying out activities associated with Vaccination? (*Please* *check all that apply)*

- Characteristics of individuals associated with your organization involved in polio eradication activities (1)
- Organizational settings (factors related to your organization supporting the polio eradication program) (2)
- Polio eradication program characteristics (the activity that was used towards eradicating polio) (3)
- Process of conducting the activities (how the activity was implemented, including the planning, execution strategies, reflection and evaluation of activities, or adjustments made to the plan) (4)
- External settings (political, economic, social, technological or environmental settings) (5)

Display This Question:

If Which of the following best describes where you experienced significant challenges when carrying... = Characteristics of individuals associated with your organization involved in polio eradication activities

Q8.6 What characteristic(s) best describes implementation challenges to Vaccination you encountered related to an **individual’s characteristics**? (*Please* *check all that apply)*

- The person’s knowledge and beliefs about the activity (individuals did not have a positive attitude towards the polio eradication activity, were unfamiliar with facts, truths and principles related to the intervention) (1)
- Self-efficacy (individuals did not have belief in own abilities to execute courses of action to achieve polio eradication goals) (2)
- Individual stage of change (how likely the individual is towards skilled, enthusiastic and sustained support of the polio eradication activities.) (3)
- Individual identification with organization (poor perception of the organization and poor relationship/commitment to the organization) (4)
- Other personal attributes (please describe): (5) ________________________________________________

Display This Question:

If Which of the following best describes where you experienced significant challenges when carrying... = Organizational settings (factors related to your organization supporting the polio eradication program)

Q8.7 What characteristic(s) best describes implementation challenges to Vaccination you encountered related to your **organizational setting**? (*Please* *check all that apply)*

- Structural characteristics (age, social architecture, size of an organization led to challenges) (1)
- Networks and communications (nature and quality of formal and informal communication in an organization led to challenges) (2)
- Culture (norms, values, basic assumptions of an organization led to challenges) (3)
- Implementation climate (capacity for change, receptivity of team to proposed intervention, relative priority of project, organizational goals, incentive and rewards, etc. led to challenges) (4)
- Readiness for implementation (Level of leadership engagement, available resources, access to knowledge and information led to challenges) (5)
- Other personal attributes (please describe): (6) ________________________________________________

Display This Question:

If Which of the following best describes where you experienced significant challenges when carrying... = Polio eradication program characteristics (the activity that was used towards eradicating polio)

Q8.8 What characteristic(s) best capture implementation challenges to Vaccination you encountered related to **GPEI program characteristics**? (*Please* *check all that apply)*

- Intervention source (perception that intervention developed either internally or externally led to challenges) (1)
- Evidence strength and quality (perceptions of the quality and validity of evidence did not support belief that the intervention will have desired outcomes) (2)
- Relative advantage (perceptions that there was another, better approach) (3)
- Adaptability to local context (The activity was not adapted, tailored or refined to meet local needs) (4)
- Trialability (no ability to test on a small scale and reverse course if warranted) (5)
- Complexity (perceived difficulty of implementation reflected by duration, scope, radicalness, disruptiveness, centrality, and intricacy and number of steps required to implement) (6)
- Design Quality and Packaging (difficulty arising from how the intervention is bundled, presented, and assembled) (7)
- Cost (including cost of intervention, cost of implementing intervention, including investment, supply, and opportunity costs) (8)
- Other (please describe): (9) ________________________________________________

Display This Question:

If Which of the following best describes where you experienced significant challenges when carrying... = Process of conducting the activities (how the activity was implemented, including the planning, execution strategies, reflection and evaluation of activities, or adjustments made to the plan)

Q8.9 What stage(s) of the **process of program implementation** did you experience implementation challenges to Vaccination? (*Please* *check all that apply)*

- Planning (degree to which schemes/methods of implementing activities are developed in advance or poor quality of such methods) (1)
- Engaging (attracting and involving appropriate individuals or organizations in implementation of polio eradication activities) (2)
- Executing (carrying out activities according to plan) (3)
- Reflecting and evaluating (monitoring and feedback about the progress and quality of implementation accompanied with regular personal and team debriefing about progress and experience) (4)
- Other (please describe): (5) ________________________________________________

Display This Question:

If Which of the following best describes where you experienced significant challenges when carrying... = External settings (political, economic, social, technological or environmental settings)

Q8.10 What characteristic(s) best capture implementation challenges of Vaccination you encountered related to **external settings**? (*Please* *check all that apply)*

- Political environment (Policymaker disinterest or resistance, limited windows of opportunity within the political climate, political structure non-conducive to coordinated action.) (1)
- Economic environment (Insufficient revenue sources/base to fund activities and/or maintain system developments) (2)
- Social environment (Communities in which polio eradication activities were implemented are non-accepting and/or resistant to intervention) (3)
- Technological environment (Slow or limited advances of technologies used in implementing polio eradication activities) (4)
- Other environment (environment where activity was implemented was prohibitive and did not contribute to the success of polio eradication, including the global climate and ineffective cross-organizational collaboration) (5)

Q8.11 Please describe the most influential internal, external or combination of challenges you experienced when carrying out activities associated with Vaccination

________________________________________________________________

________________________________________________________________

________________________________________________________________

________________________________________________________________

________________________________________________________________

End of Block: Vaccination

Start of Block: Surveillance

Q9.1 *For this next set of questions, we would like to ask you about implementation facilitators and challenges relevant to the objective of* ***Surveillance***

Q9.2 In your opinion, which of the following was the biggest ***internal*** ***contributor*** to your program's success in completing its objective of Surveillance*?*

- Characteristics of individuals within your organization involved in polio eradication activities (1)
- Organizational settings (factors related to your organization supporting the polio eradication program) (2)
- Polio eradication program characteristics (the activity that was used towards eradicating polio, including technologies that were adopted by the organization/individual implementing the activities) (3)
- Process of conducting the activities (how the activity was implemented, including the planning, execution strategies, reflection and evaluation of activities, or adjustments made to the plan) (4)

Q9.3 In your opinion, which of the following was the biggest ***external contributor*** to your program’s success in completing its objective of Surveillance*?*

- Political environment (lawmaker support, political climate accepting of polio eradication activities, and political structure to conducive to coordinated action) (1)
- Economic environment (sufficient revenue sources/base to fund activities and/or maintain system developments) (2)
- Social environment (social norms around immunization, accepting communities in which polio eradication activities were implemented) (3)
- Technological environment (infrastructure or technological advances outside of the organization) (4)
- Other environment (Please describe): (5) ________________________________________________

Q9.4 Please briefly describe the most influential internal, external or combination of contributors to your program's success in completing its objective of Surveillance

________________________________________________________________

________________________________________________________________

________________________________________________________________

________________________________________________________________

________________________________________________________________

Q9.5 Which of the following best describes where you experienced significant ***challenges*** when carrying out activities associated with Surveillance? (*Please* *check all that apply)*

- Characteristics of individuals associated with your organization involved in polio eradication activities (1)
- Organizational settings (factors related to your organization supporting the polio eradication program) (2)
- Polio eradication program characteristics (the activity that was used towards eradicating polio) (3)
- Process of conducting the activities (how the activity was implemented, including the planning, execution strategies, reflection and evaluation of activities, or adjustments made to the plan) (4)
- External settings (political, economic, social, technological or environmental settings) (5)

Display This Question:

If Which of the following best describes where you experienced significant challenges when carrying... = Characteristics of individuals associated with your organization involved in polio eradication activities

Q9.6 What characteristic(s) best describes implementation challenges to Surveillance you encountered related to an **individual’s characteristics**? (*Please* *check all that apply)*

- The person’s knowledge and beliefs about the activity (individuals did not have a positive attitude towards the polio eradication activity, were unfamiliar with facts, truths and principles related to the intervention) (1)
- Self-efficacy (individuals did not have belief in own abilities to execute courses of action to achieve polio eradication goals) (2)
- Individual stage of change (how likely the individual is towards skilled, enthusiastic and sustained support of the polio eradication activities.) (3)
- Individual identification with organization (poor perception of the organization and poor relationship/commitment to the organization) (4)
- Other personal attributes (please describe): (5) ________________________________________________

Display This Question:

If Which of the following best describes where you experienced significant challenges when carrying... = Organizational settings (factors related to your organization supporting the polio eradication program)

Q9.7 What characteristic(s) best describes implementation challenges to Surveillance you encountered related to your **organizational setting**? (*Please* *check all that apply)*

- Structural characteristics (age, social architecture, size of an organization led to challenges) (1)
- Networks and communications (nature and quality of formal and informal communication in an organization led to challenges) (2)
- Culture (norms, values, basic assumptions of an organization led to challenges) (3)
- Implementation climate (capacity for change, receptivity of team to proposed intervention, relative priority of project, organizational goals, incentive and rewards, etc. led to challenges) (4)
- Readiness for implementation (Level of leadership engagement, available resources, access to knowledge and information led to challenges) (5)
- Other personal attributes (please describe): (6) ________________________________________________

Display This Question:

If Which of the following best describes where you experienced significant challenges when carrying... = Polio eradication program characteristics (the activity that was used towards eradicating polio)

Q9.8 What characteristic(s) best capture implementation challenges to Surveillance you encountered related to **GPEI program characteristics**? (*Please* *check all that apply)*

- Intervention source (perception that intervention developed either internally or externally led to challenges) (1)
- Evidence strength and quality (perceptions of the quality and validity of evidence did not support belief that the intervention will have desired outcomes) (2)
- Relative advantage (perceptions that there was another, better approach) (3)
- Adaptability to local context (The activity was not adapted, tailored or refined to meet local needs) (4)
- Trialability (no ability to test on a small scale and reverse course if warranted) (5)
- Complexity (perceived difficulty of implementation reflected by duration, scope, radicalness, disruptiveness, centrality, and intricacy and number of steps required to implement) (6)
- Design Quality and Packaging (difficulty arising from how the intervention is bundled, presented, and assembled) (7)
- Cost (including cost of intervention, cost of implementing intervention, including investment, supply, and opportunity costs) (8)
- Other (please describe): (9) ________________________________________________

Display This Question:

If Which of the following best describes where you experienced significant challenges when carrying... = Process of conducting the activities (how the activity was implemented, including the planning, execution strategies, reflection and evaluation of activities, or adjustments made to the plan)

Q9.9 What stage(s) of the **process of program implementation** did you experience implementation challenges to Surveillance? (*Please* *check all that apply)*

- Planning (degree to which schemes/methods of implementing activities are developed in advance or poor quality of such methods) (1)
- Engaging (attracting and involving appropriate individuals or organizations in implementation of polio eradication activities) (2)
- Executing (carrying out activities according to plan) (3)
- Reflecting and evaluating (monitoring and feedback about the progress and quality of implementation accompanied with regular personal and team debriefing about progress and experience) (4)
- Other (please describe): (5) ________________________________________________

Display This Question:

If Which of the following best describes where you experienced significant challenges when carrying... = External settings (political, economic, social, technological or environmental settings)

Q9.10 What characteristic(s) best capture implementation challenges of Surveillance you encountered related to **external settings**? (*Please* *check all that apply)*

- Political environment (Policymaker disinterest or resistance, limited windows of opportunity within the political climate, political structure non-conducive to coordinated action.) (1)
- Economic environment (Insufficient revenue sources/base to fund activities and/or maintain system developments) (2)
- Social environment (Communities in which polio eradication activities were implemented are non-accepting and/or resistant to intervention) (3)
- Technological environment (Slow or limited advances of technologies used in implementing polio eradication activities) (4)
- Other environment (environment where activity was implemented was prohibitive and did not contribute to the success of polio eradication, including the global climate and ineffective cross-organizational collaboration) (5)

Q9.11 Please describe the most influential internal, external or combination of challenges you experienced when carrying out activities associated with Surveillance

________________________________________________________________

________________________________________________________________

________________________________________________________________

________________________________________________________________

________________________________________________________________

End of Block: Surveillance

Start of Block: Community Engagement

Q10.1 *For this next set of questions, we would like to ask you about implementation facilitators and challenges relevant to the objective of* ***Community Engagement***

Q10.2 In your opinion, which of the following was the biggest ***internal*** ***contributor*** to your program's success in completing its objective of Community Engagement*?*

- Characteristics of individuals within your organization involved in polio eradication activities (1)
- Organizational settings (factors related to your organization supporting the polio eradication program) (2)
- Polio eradication program characteristics (the activity that was used towards eradicating polio, including technologies that were adopted by the organization/individual implementing the activities) (3)
- Process of conducting the activities (how the activity was implemented, including the planning, execution strategies, reflection and evaluation of activities, or adjustments made to the plan) (4)

Q10.3 In your opinion, which of the following was the biggest ***external contributor*** to your program’s success in completing its objective of Community Engagement*?*

- Political environment (lawmaker support, political climate accepting of polio eradication activities, and political structure to conducive to coordinated action) (1)
- Economic environment (sufficient revenue sources/base to fund activities and/or maintain system developments) (2)
- Social environment (social norms around immunization, accepting communities in which polio eradication activities were implemented) (3)
- Technological environment (infrastructure or technological advances outside of the organization) (4)
- Other environment (Please describe): (5) ________________________________________________

Q10.4 Please briefly describe the most influential internal, external or combination of contributors to your program's success in completing its objective of Community Engagement

________________________________________________________________

________________________________________________________________

________________________________________________________________

________________________________________________________________

________________________________________________________________

Q10.5 Which of the following best describes where you experienced significant ***challenges*** when carrying out activities associated with Community Engagement? (*Please* *check all that apply)*

- Characteristics of individuals associated with your organization involved in polio eradication activities (1)
- Organizational settings (factors related to your organization supporting the polio eradication program) (2)
- Polio eradication program characteristics (the activity that was used towards eradicating polio) (3)
- Process of conducting the activities (how the activity was implemented, including the planning, execution strategies, reflection and evaluation of activities, or adjustments made to the plan) (4)
- External settings (political, economic, social, technological or environmental settings) (5)

Display This Question:

If Which of the following best describes where you experienced significant challenges when carrying... = Characteristics of individuals associated with your organization involved in polio eradication activities

Q10.6 What characteristic(s) best describes implementation challenges to Community Engagement you encountered related to an **individual’s characteristics**? (*Please* *check all that apply)*

- The person’s knowledge and beliefs about the activity (individuals did not have a positive attitude towards the polio eradication activity, were unfamiliar with facts, truths and principles related to the intervention) (1)
- Self-efficacy (individuals did not have belief in own abilities to execute courses of action to achieve polio eradication goals) (2)
- Individual stage of change (how likely the individual is towards skilled, enthusiastic and sustained support of the polio eradication activities.) (3)
- Individual identification with organization (poor perception of the organization and poor relationship/commitment to the organization) (4)
- Other personal attributes (please describe): (5) ________________________________________________

Display This Question:

If Which of the following best describes where you experienced significant challenges when carrying... = Organizational settings (factors related to your organization supporting the polio eradication program)

Q10.7 What characteristic(s) best describes implementation challenges to Community Engagement you encountered related to your **organizational setting**? (*Please* *check all that apply)*

- Structural characteristics (age, social architecture, size of an organization led to challenges) (1)
- Networks and communications (nature and quality of formal and informal communication in an organization led to challenges) (2)
- Culture (norms, values, basic assumptions of an organization led to challenges) (3)
- Implementation climate (capacity for change, receptivity of team to proposed intervention, relative priority of project, organizational goals, incentive and rewards, etc. led to challenges) (4)
- Readiness for implementation (Level of leadership engagement, available resources, access to knowledge and information led to challenges) (5)
- Other personal attributes (please describe): (6) ________________________________________________

Display This Question:

If Which of the following best describes where you experienced significant challenges when carrying... = Polio eradication program characteristics (the activity that was used towards eradicating polio)

Q10.8 What characteristic(s) best capture implementation challenges to Community Engagement you encountered related to **GPEI program characteristics**? (*Please* *check all that apply)*

- Intervention source (perception that intervention developed either internally or externally led to challenges) (1)
- Evidence strength and quality (perceptions of the quality and validity of evidence did not support belief that the intervention will have desired outcomes) (2)
- Relative advantage (perceptions that there was another, better approach) (3)
- Adaptability to local context (The activity was not adapted, tailored or refined to meet local needs) (4)
- Trialability (no ability to test on a small scale and reverse course if warranted) (5)
- Complexity (perceived difficulty of implementation reflected by duration, scope, radicalness, disruptiveness, centrality, and intricacy and number of steps required to implement) (6)
- Design Quality and Packaging (difficulty arising from how the intervention is bundled, presented, and assembled) (7)
- Cost (including cost of intervention, cost of implementing intervention, including investment, supply, and opportunity costs) (8)
- Other (please describe): (9) ________________________________________________

Display This Question:

If Which of the following best describes where you experienced significant challenges when carrying... = Process of conducting the activities (how the activity was implemented, including the planning, execution strategies, reflection and evaluation of activities, or adjustments made to the plan)

Q10.9 What stage(s) of the **process of program implementation** did you experience implementation challenges to Community Engagement? (*Please* *check all that apply)*

- Planning (degree to which schemes/methods of implementing activities are developed in advance or poor quality of such methods) (1)
- Engaging (attracting and involving appropriate individuals or organizations in implementation of polio eradication activities) (2)
- Executing (carrying out activities according to plan) (3)
- Reflecting and evaluating (monitoring and feedback about the progress and quality of implementation accompanied with regular personal and team debriefing about progress and experience) (4)
- Other (please describe): (5) ________________________________________________

Display This Question:

If Which of the following best describes where you experienced significant challenges when carrying... = External settings (political, economic, social, technological or environmental settings)

Q10.10 What characteristic(s) best capture implementation challenges of Community Engagement you encountered related to **external settings**? (*Please* *check all that apply)*

- Political environment (Policymaker disinterest or resistance, limited windows of opportunity within the political climate, political structure non-conducive to coordinated action.) (1)
- Economic environment (Insufficient revenue sources/base to fund activities and/or maintain system developments) (2)
- Social environment (Communities in which polio eradication activities were implemented are non-accepting and/or resistant to intervention) (3)
- Technological environment (Slow or limited advances of technologies used in implementing polio eradication activities) (4)
- Other environment (environment where activity was implemented was prohibitive and did not contribute to the success of polio eradication, including the global climate and ineffective cross-organizational collaboration) (5)

Q10.11 Please describe the most influential internal, external or combination of challenges you experienced when carrying out activities associated with Community Engagement

________________________________________________________________

________________________________________________________________

________________________________________________________________

________________________________________________________________

________________________________________________________________

End of Block: Community Engagement

Start of Block: Monitoring and Evaluation

Q11.1 *For this next set of questions, we would like to ask you about implementation facilitators and challenges relevant to the objective of* ***Monitoring and Evaluation***

Q11.2 In your opinion, which of the following was the biggest ***internal*** ***contributor*** to your program's success in completing its objective of Monitoring and Evaluation*?*

- Characteristics of individuals within your organization involved in polio eradication activities (1)
- Organizational settings (factors related to your organization supporting the polio eradication program) (2)
- Polio eradication program characteristics (the activity that was used towards eradicating polio, including technologies that were adopted by the organization/individual implementing the activities) (3)
- Process of conducting the activities (how the activity was implemented, including the planning, execution strategies, reflection and evaluation of activities, or adjustments made to the plan) (4)

Q11.3 In your opinion, which of the following was the biggest ***external contributor*** to your program’s success in completing its objective of Monitoring and Evaluation*?*

- Political environment (lawmaker support, political climate accepting of polio eradication activities, and political structure to conducive to coordinated action) (1)
- Economic environment (sufficient revenue sources/base to fund activities and/or maintain system developments) (2)
- Social environment (social norms around immunization, accepting communities in which polio eradication activities were implemented) (3)
- Technological environment (infrastructure or technological advances outside of the organization) (4)
- Other environment (Please describe): (5) ________________________________________________

Q11.4 Please briefly describe the most influential internal, external or combination of contributors to your program's success in completing its objective of Monitoring and Evaluation

________________________________________________________________

________________________________________________________________

________________________________________________________________

________________________________________________________________

________________________________________________________________

Q11.5 Which of the following best describes where you experienced significant ***challenges*** when carrying out activities associated with Monitoring and Evaluation? (*Please* *check all that apply)*

- Characteristics of individuals associated with your organization involved in polio eradication activities (1)
- Organizational settings (factors related to your organization supporting the polio eradication program) (2)
- Polio eradication program characteristics (the activity that was used towards eradicating polio) (3)
- Process of conducting the activities (how the activity was implemented, including the planning, execution strategies, reflection and evaluation of activities, or adjustments made to the plan) (4)
- External settings (political, economic, social, technological or environmental settings) (5)

Display This Question:

If Which of the following best describes where you experienced significant challenges when carrying... = Characteristics of individuals associated with your organization involved in polio eradication activities

Q11.6 What characteristic(s) best describes implementation challenges to Monitoring and Evaluation you encountered related to an **individual’s characteristics**? (*Please* *check all that apply)*

- The person’s knowledge and beliefs about the activity (individuals did not have a positive attitude towards the polio eradication activity, were unfamiliar with facts, truths and principles related to the intervention) (1)
- Self-efficacy (individuals did not have belief in own abilities to execute courses of action to achieve polio eradication goals) (2)
- Individual stage of change (how likely the individual is towards skilled, enthusiastic and sustained support of the polio eradication activities.) (3)
- Individual identification with organization (poor perception of the organization and poor relationship/commitment to the organization) (4)
- Other personal attributes (please describe): (5) ________________________________________________

Display This Question:

If Which of the following best describes where you experienced significant challenges when carrying... = Organizational settings (factors related to your organization supporting the polio eradication program)

Q11.7 What characteristic(s) best describes implementation challenges to Monitoring and Evaluation you encountered related to your **organizational setting**? (*Please* *check all that apply)*

- Structural characteristics (age, social architecture, size of an organization led to challenges) (1)
- Networks and communications (nature and quality of formal and informal communication in an organization led to challenges) (2)
- Culture (norms, values, basic assumptions of an organization led to challenges) (3)
- Implementation climate (capacity for change, receptivity of team to proposed intervention, relative priority of project, organizational goals, incentive and rewards, etc. led to challenges) (4)
- Readiness for implementation (Level of leadership engagement, available resources, access to knowledge and information led to challenges) (5)
- Other personal attributes (please describe): (6) ________________________________________________

Display This Question:

If Which of the following best describes where you experienced significant challenges when carrying... = Polio eradication program characteristics (the activity that was used towards eradicating polio)

Q11.8 What characteristic(s) best capture implementation challenges to Monitoring and Evaluation you encountered related to **GPEI program characteristics**? (*Please* *check all that apply)*

- Intervention source (perception that intervention developed either internally or externally led to challenges) (1)
- Evidence strength and quality (perceptions of the quality and validity of evidence did not support belief that the intervention will have desired outcomes) (2)
- Relative advantage (perceptions that there was another, better approach) (3)
- Adaptability to local context (The activity was not adapted, tailored or refined to meet local needs) (4)
- Trialability (no ability to test on a small scale and reverse course if warranted) (5)
- Complexity (perceived difficulty of implementation reflected by duration, scope, radicalness, disruptiveness, centrality, and intricacy and number of steps required to implement) (6)
- Design Quality and Packaging (difficulty arising from how the intervention is bundled, presented, and assembled) (7)
- Cost (including cost of intervention, cost of implementing intervention, including investment, supply, and opportunity costs) (8)
- Other (please describe): (9) ________________________________________________

Display This Question:

If Which of the following best describes where you experienced significant challenges when carrying... = Process of conducting the activities (how the activity was implemented, including the planning, execution strategies, reflection and evaluation of activities, or adjustments made to the plan)

Q11.9 What stage(s) of the **process of program implementation** did you experience implementation challenges to Monitoring and Evaluation? (*Please* *check all that apply)*

- Planning (degree to which schemes/methods of implementing activities are developed in advance or poor quality of such methods) (1)
- Engaging (attracting and involving appropriate individuals or organizations in implementation of polio eradication activities) (2)
- Executing (carrying out activities according to plan) (3)
- Reflecting and evaluating (monitoring and feedback about the progress and quality of implementation accompanied with regular personal and team debriefing about progress and experience) (4)
- Other (please describe): (5) ________________________________________________

Display This Question:

If Which of the following best describes where you experienced significant challenges when carrying... = External settings (political, economic, social, technological or environmental settings)

Q11.10 What characteristic(s) best capture implementation challenges of Monitoring and Evaluation you encountered related to **external settings**? (*Please* *check all that apply)*

- Political environment (Policymaker disinterest or resistance, limited windows of opportunity within the political climate, political structure non-conducive to coordinated action.) (1)
- Economic environment (Insufficient revenue sources/base to fund activities and/or maintain system developments) (2)
- Social environment (Communities in which polio eradication activities were implemented are non-accepting and/or resistant to intervention) (3)
- Technological environment (Slow or limited advances of technologies used in implementing polio eradication activities) (4)
- Other environment (environment where activity was implemented was prohibitive and did not contribute to the success of polio eradication, including the global climate and ineffective cross-organizational collaboration) (5)

Q11.11 Please describe the most influential internal, external or combination of challenges you experienced when carrying out activities associated with Monitoring and Evaluation

________________________________________________________________

________________________________________________________________

________________________________________________________________

________________________________________________________________

________________________________________________________________

End of Block: Monitoring and Evaluation

Start of Block: Other

Q12.1 *For this next set of questions, we would like to ask you about implementation facilitators and challenges relevant to the objective of ${Q3.2/ChoiceTextEntryValue/9}*

Q12.2 In your opinion, which of the following was the biggest ***internal*** ***contributor*** to your program's success in completing its objective of ${Q3.2/ChoiceTextEntryValue/9}*?*

- Characteristics of individuals within your organization involved in polio eradication activities (1)
- Organizational settings (factors related to your organization supporting the polio eradication program) (2)
- Polio eradication program characteristics (the activity that was used towards eradicating polio, including technologies that were adopted by the organization/individual implementing the activities) (3)
- Process of conducting the activities (how the activity was implemented, including the planning, execution strategies, reflection and evaluation of activities, or adjustments made to the plan) (4)

Q12.3 In your opinion, which of the following was the biggest ***external contributor*** to your program’s success in completing its objective of ${Q3.2/ChoiceTextEntryValue/9}*?*

- Political environment (lawmaker support, political climate accepting of polio eradication activities, and political structure to conducive to coordinated action) (1)
- Economic environment (sufficient revenue sources/base to fund activities and/or maintain system developments) (2)
- Social environment (social norms around immunization, accepting communities in which polio eradication activities were implemented) (3)
- Technological environment (infrastructure or technological advances outside of the organization) (4)
- Other environment (Please describe): (5) ________________________________________________

Q12.4 Please briefly describe the most influential internal, external or combination of contributors to your program's success in completing its objective of ${Q3.2/ChoiceTextEntryValue/9}

________________________________________________________________

________________________________________________________________

________________________________________________________________

________________________________________________________________

________________________________________________________________

Q12.5 Which of the following best describes where you experienced significant ***challenges*** when carrying out activities associated with ${Q3.2/ChoiceTextEntryValue/9}? (*Please* *check all that apply)*

- Characteristics of individuals associated with your organization involved in polio eradication activities (1)
- Organizational settings (factors related to your organization supporting the polio eradication program) (2)
- Polio eradication program characteristics (the activity that was used towards eradicating polio) (3)
- Process of conducting the activities (how the activity was implemented, including the planning, execution strategies, reflection and evaluation of activities, or adjustments made to the plan) (4)
- External settings (political, economic, social, technological or environmental settings) (5)

Display This Question:

If Which of the following best describes where you experienced significant challenges when carrying... = Characteristics of individuals associated with your organization involved in polio eradication activities

Q12.6 What characteristic(s) best describes implementation challenges to ${Q3.2/ChoiceTextEntryValue/9} you encountered related to an **individual’s characteristics**? (*Please* *check all that apply)*

- The person’s knowledge and beliefs about the activity (individuals did not have a positive attitude towards the polio eradication activity, were unfamiliar with facts, truths and principles related to the intervention) (1)
- Self-efficacy (individuals did not have belief in own abilities to execute courses of action to achieve polio eradication goals) (2)
- Individual stage of change (how likely the individual is towards skilled, enthusiastic and sustained support of the polio eradication activities.) (3)
- Individual identification with organization (poor perception of the organization and poor relationship/commitment to the organization) (4)
- Other personal attributes (please describe): (5) ________________________________________________

Display This Question:

If Which of the following best describes where you experienced significant challenges when carrying... = Organizational settings (factors related to your organization supporting the polio eradication program)

Q12.7 What characteristic(s) best describes implementation challenges to ${Q3.2/ChoiceTextEntryValue/9} you encountered related to your **organizational setting**? (*Please* *check all that apply)*

- Structural characteristics (age, social architecture, size of an organization led to challenges) (1)
- Networks and communications (nature and quality of formal and informal communication in an organization led to challenges) (2)
- Culture (norms, values, basic assumptions of an organization led to challenges) (3)
- Implementation climate (capacity for change, receptivity of team to proposed intervention, relative priority of project, organizational goals, incentive and rewards, etc. led to challenges) (4)
- Readiness for implementation (Level of leadership engagement, available resources, access to knowledge and information led to challenges) (5)
- Other personal attributes (please describe): (6) ________________________________________________

Display This Question:

If Which of the following best describes where you experienced significant challenges when carrying... = Polio eradication program characteristics (the activity that was used towards eradicating polio)

Q12.8 What characteristic(s) best capture implementation challenges to ${Q3.2/ChoiceTextEntryValue/9} you encountered related to **GPEI program characteristics**? (*Please* *check all that apply)*

- Intervention source (perception that intervention developed either internally or externally led to challenges) (1)
- Evidence strength and quality (perceptions of the quality and validity of evidence did not support belief that the intervention will have desired outcomes) (2)
- Relative advantage (perceptions that there was another, better approach) (3)
- Adaptability to local context (The activity was not adapted, tailored or refined to meet local needs) (4)
- Trialability (no ability to test on a small scale and reverse course if warranted) (5)
- Complexity (perceived difficulty of implementation reflected by duration, scope, radicalness, disruptiveness, centrality, and intricacy and number of steps required to implement) (6)
- Design Quality and Packaging (difficulty arising from how the intervention is bundled, presented, and assembled) (7)
- Cost (including cost of intervention, cost of implementing intervention, including investment, supply, and opportunity costs) (8)
- Other (please describe): (9) ________________________________________________

Display This Question:

If Which of the following best describes where you experienced significant challenges when carrying... = Process of conducting the activities (how the activity was implemented, including the planning, execution strategies, reflection and evaluation of activities, or adjustments made to the plan)

Q12.9 What stage(s) of the **process of program implementation** did you experience implementation challenges to ${Q3.2/ChoiceTextEntryValue/9}? (*Please* *check all that apply)*

- Planning (degree to which schemes/methods of implementing activities are developed in advance or poor quality of such methods) (1)
- Engaging (attracting and involving appropriate individuals or organizations in implementation of polio eradication activities) (2)
- Executing (carrying out activities according to plan) (3)
- Reflecting and evaluating (monitoring and feedback about the progress and quality of implementation accompanied with regular personal and team debriefing about progress and experience) (4)
- Other (please describe): (5) ________________________________________________

Display This Question:

If Which of the following best describes where you experienced significant challenges when carrying... = External settings (political, economic, social, technological or environmental settings)

Q12.10 What characteristic(s) best capture implementation challenges of ${Q3.2/ChoiceTextEntryValue/9} you encountered related to **external settings**? (*Please* *check all that apply)*

- Political environment (Policymaker disinterest or resistance, limited windows of opportunity within the political climate, political structure non-conducive to coordinated action.) (1)
- Economic environment (Insufficient revenue sources/base to fund activities and/or maintain system developments) (2)
- Social environment (Communities in which polio eradication activities were implemented are non-accepting and/or resistant to intervention) (3)
- Technological environment (Slow or limited advances of technologies used in implementing polio eradication activities) (4)
- Other environment (environment where activity was implemented was prohibitive and did not contribute to the success of polio eradication, including the global climate and ineffective cross-organizational collaboration) (5)

Q12.11 Please describe the most influential internal, external or combination of challenges you experienced when carrying out activities associated with ${Q3.2/ChoiceTextEntryValue/9}

________________________________________________________________

________________________________________________________________

________________________________________________________________

________________________________________________________________

________________________________________________________________

End of Block: Other

Start of Block: Unintended Consequences

Q13.1 Unintended consequences are any outcomes of GPEI-related activities that were not associated with the expected results of the work and may be both positive or negative in nature.   Examples of unintended consequences may include: GPEI activities that contributed to delivery of non-immunization health services, improved performance management of health services, displaced health workers from non-immunization health services, impacted social determinants of health such as housing, water infrastructure, or contributed to trust or mistrust of government and government supported services.

 Please briefly describe any unintended consequences you experienced during your involvement with GPEI activities.

________________________________________________________________

________________________________________________________________

________________________________________________________________

________________________________________________________________

________________________________________________________________

End of Block: Unintended Consequences

Start of Block: Follow Up

Q14.1 We are trying to capture a comprehensive set of experiences with the GPEI, including the experiences of frontline implementers whose perceptions of lessons learned may be undocumented.

Q14.2 Do you have contact information for any frontline workers associated with GPEI projects that you would be willing to share?

- Yes (1)
- No (2)

Skip To: Q14.5 If Do you have contact information for any frontline workers associated with GPEI projects that you... = No

Q14.3 Please enter names and contact information for frontline workers:

________________________________________________________________

________________________________________________________________

________________________________________________________________

________________________________________________________________

________________________________________________________________

Q14.4  In your opinion, what is the best way to reach these front-line workers with this survey?

- Send an online survey in English (1)
- Send an online survey in (national language logic based on answer to demographic question of where they did GPEI work) (2)
- Interview administered by mobile phone (3)
- In-person interview (4)

| Page Break |  |
| --- | --- |

Q14.5 We are conducting in-depth interviews with individuals who are identified as key actors who resolved GPEI implementation challenges at the national, subnational and field levels.   Would you like to be considered for an in-depth interview to capture implementation issues?

- Yes (1)
- No (2)

Skip To: Q14.7 If We are conducting in-depth interviews with individuals who are identified as key actors who resol... = No

Q14.6 Please provide your contact information so that we can reach you at a later date:

________________________________________________________________

Q14.7 Would you like to recommend someone else to be considered for an in-depth interview to capture implementation issues?

- Yes (1)
- No (2)

Q14.8 Please list the person's name, contact information, and a brief explanation why you think this person should be considered for an in-depth interview

- Candidate's name (1) ________________________________________________
- Candidate's contact information (2) ________________________________________________
- Reason for nomination (3) ________________________________________________

| Page Break |  |
| --- | --- |

Q14.9 Thank you for your time spent answering this survey and for your contributions to the efforts to eradicate polio globally.


 Should you have any additional questions or concerns about this study or wish to nominate a respondent at a later date, please reach out to the project team at poliolessonslearned@gmail.com.

End of Block: Follow Up
