# Supplementary material for: Synthesis and translation of research and innovations from polio eradication (STRIPE): initial findings from a global mixed methods study
Source: BMC Public Health. 2020 Aug 12;20(Suppl 2):1176. doi: 10.1186/s12889-020-09156-9 (PMC7421832; doi:10.1186/s12889-020-09156-9)
Supplement: Supplementary file 2 — Additional file 2. [file 12889_2020_9156_MOESM2_ESM.docx]

**Appendix II**

**Merged KII Tool | National / Sub-national Actors**

*[Obtain consent to conduct the interview]*

*Thank you for meeting with me today.*

*We're interested in learning about your experiences with polio eradication activities that you have led or been a part of. Specifically, we want to know about what occurred, what worked, what didn’t work, the challenges you faced while doing this work, and how you were able to tackle these challenges, as well as what you learned and think others could learn.*

*The questions I am going to ask don't have right or wrong answers. This conversation is completely confidential, and we can skip any question that you prefer not to answer.*

*Is it okay if I audio record our conversation today, for purposes of transcription and documentation? It will not be shared with anyone outside the research team.*

**I. Demographics**

- Age
- Gender
- Current affiliation and designation
- How did you become involved in global polio eradication activities?
  - What kind of work have you done with the polio initiative?
- Tell me a little about the polio program in [**country**].

**II. Polio program organization and change**

*We’d first like to understand how the polio program was organized in [****country****], what the program did and who was involved.*

1. How was the polio program organized in [**country**]?

- How did the organization differ (if at all) from how the national immunization program was structured?
- How did the polio program differ from how other health services were delivered, such as maternal and child care, malaria prevention, WASH programs, etc.?
- Was this structure consistent across [**national/subnational/etc.**] levels?

1. Did the structure of the polio program change over time? If so, how?
   - *For example:* accountability structures, staffing, who paid for activities, etc.
2. For the work you did related to the polio program, how did your work in polio eradication **support or differ** from other health programs you were involved in?
   - How did your work activities support or differ from the national immunization program?
   - *[If worked at subnational level]* How did your work activities support or differ from the national polio eradication activities?
   - *Probe for all sub-questions:* How was it delivered? Were personnel differentiated? Hours of work, pay. Who was managing it?
   - How (if at all) did the polio program make your work harder or easier?
3. What changes (if any) in activities occurred over the course of the polio eradication program?
   - *Probe: your role, your daily tasks, your location, who you worked with, who you reported to etc.*
   - *Probe:* *(If not obvious)* When did these changes occur? Why do you think these changes occurred? What resulted from these changes?
4. What changes (if any) in the key actors, either implementers or funders, over the course of the polio eradication program?
   - *Probe:* *(If not obvious)* When did these changes occur? Why do you think these changes occurred? What resulted from these changes?

**III. Context and Challenges**

*Next, we’d like to understand how the environment (including the political, social, economic, technical and legal environment) and the health system (including health financing, information, workforce, supplies, governance and service delivery) may have affected or been affected by the polio eradication program. We are interested in knowing about the positive ways in which the programs/systems might have worked together, as well as ways in which one may have hindered the other. For these questions, we’d also like to understand what challenges you faced in your work at a personal, organizational or systems level.*

1. Were there any major factors (political, social, economic, technical, environmental, legal) and/or changes in the country that affected the implementation of the polio eradication program? How was implementation affected? *(for example, a supportive government, lack of infrastructure, development of a new technology, new political leadership, an earthquake, inflation, conflict, etc.)*
   - *Probe:* How did these changes *support* or *hinder* implementation?
   - *Probe:* Were these unexpected? Did they cause any deviations, additions, setbacks or changes to the program?
   - *Probe:* Did you have to adapt your work in anyway (personally, or as an organization)? If no solutions were developed, why do you think that was?
2. Were there any health system factors that affected implementation of the polio program? For example, changes to service delivery, health workforce shortages. How did these factors affect implementation?
   - *Probe:* How did these factors *support* or *hinder* implementation?
   - *Probe:* Were these effects unexpected? Did they cause any deviations, additions, setbacks or changes to the program?
   - *Probe:* Did you have to adapt your work in anyway (personally, or as an organization)? If no solutions were developed, why do you think that was?
3. Conversely, did carrying out any polio eradication activities bring about changes in the country’s political, social, economic, technical or legal environment, or to the health system? What about changes to the national immunization program? Other health programs?
   - *Probe:* Were these changes positive or negative?
   - *Probe:* Did they shift over time?

*[Respondents may naturally bring up challenges related to the environment and/or health system in their answers to questions 5-7. If they do, then proceed with question 8a. If challenges have not been previously discussed, proceed with question 8b.]*

***[If challenges discussed]***

8a. Earlier, you talked about some of the challenges faced, can you talk about some of the specific challenges you faced (personally, interpersonally, institutionally) in carrying out polio eradication activities?

- - *Probe:* How did you or your team deal with these challenges?
  - Were any of these challenges related to gender or gender dynamics? If yes, please explain.
  - *Probe***:** Were there any gender differences in access to education, benefits, or other resources? Was there a difference in division of labor? Was there a difference in decision-making?
  - In what ways (if at all) did you have to adapt your work as a result of these challenges (personally, or as an organization)?
  - If no solutions were developed, why do you think that was?

***[If challenges not discussed]***

8b. I would like to shift now to understanding what challenges you faced either personally, interpersonally or at an organizational or institutional level. Can you talk about some of the specific challenges you faced in carrying out polio eradication activities?

- - *Probe:* How did you or your team deal with these challenges?
  - Were any of these challenges related to gender or gender dynamics? If yes, please explain.
  - *Probe***:** Were there any gender differences in access to education, benefits, or other resources? Was there a difference in division of labor? Was there a difference in decision-making?
  - In what ways (if at all) did you have to adapt your work as a result of these challenges (personally, or as an organization)?
  - If no solutions were developed, why do you think that was?
  - *Probe: [Depending on response, probe for each level]* How about personally? Interpersonally? Within your organization or institution?

1. ***[If not discussed]*** How has introduction of IPV (occurred or not) affected polio eradication activities in country?
   - Delays in introduction? Availability issues? How did you find out? How did you change your plans?
2. Of all the challenges you described (at the country level, personally, etc.), which do you think was the greatest challenge and why?

**IV. Strategies and Solutions**

*We’re really interested in learning about innovative solutions that were tried and learned to address implementation challenges, during you work in polio eradication.*

*[In Section III above, interviewer should make a bulleted list of challenges enumerated and refer to each of those challenges in asking question 11.]*

1. Earlier you mentioned a series of challenges *(interviewer list challenges noted by respondent) -* can you say a little about any strategies you and your team developed to address these challenges?
   - *Probe:* Who were involved in developing these solutions?

Did it work?

- - *Probe:* How do you know it worked or didn’t work? Did they bring about any positive or negative change?

How did people react to these changes?

- - *Probe:* e.g. Resistance? Acceptance?

Did it result in any changes based on these solutions?

- - *Probe:* e.g. change in protocol or operational procedures

What would have made it easier to tackle this challenge?

- - *Probe:* more people, a better vaccine delivery system to avoid stock outs, a more responsive government, etc.

1. What other strategies did you try? Did they work or not? Why?

**V. Lessons Learned**

*One important goal of this research is translating knowledge learned from the polio eradication effort so that it may be used in the future for other healthcare efforts.*

1. In your experience, how have assets and/or contributions *(for example s*kills, tools, infrastructure, people etc.) from the polio initiative been useful for other health efforts?
   1. *Probe:* e.g. in some countries, there’s anecdotal evidence to suggest polio health workers became so familiar with communities and households while making their rounds, after some time they also started delivering newborn and maternal health interventions.
   2. What about in other sectors beyond health?
2. You mentioned **[X]** above, can you share any other lessons that you have learned from your work with polio eradication that you think others should know?
   1. How have you used any of these lessons in other areas of your work?
   2. Have you shared these lessons with colleagues within and outside the polio program? If so, how?
3. How else do you think lessons from polio eradication activities would be used in other areas of health? In other sectors beyond health?
   - Do you think there were any missed opportunities for applying lessons from polio to other areas?

**VI. Transition**

*Finally, we are interested in understanding any changes to the program since the polio virus has been eliminated* ***OR*** *any changes to the program you think may occur after the polio virus is eliminated. This might include changes to the work you do, efforts to utilize polio resources for other programs, changes in who is involved or manages program activities (the government, NGOs), etc.*

***[If country is polio-free]***

16a. Since [country] was declared polio free, what changes have occurred to the program?

1. Which activities are ongoing? Which have stopped? How has your job shifted, if at all?
   - *Probes: Level of pay, who employs you, who supervises you, where you work, what activities you are involved in, etc.*
2. Have you seen any changes in how immunizations, other programs are being delivered now?
3. How has this transition gone in your opinion? Do you think any key issues are being addressed, why or why not?

***[If country still has circulating wild polio virus]***

16b. Can you talk about any ongoing activities to address circulating vaccine derived poliovirus (cVDPV)?

- *Probes: continuing environmental surveillance, maintain vaccine coverage, use of IPV, issues with the supply of IPV etc.*

16c. When [country] is declared polio free, what changes to the program do you think will occur?

1. Will activities be ongoing? Will any activities stop? Do you anticipate your job will shift? If so, how?
   - *Probes: Level of pay, who employs you, who supervises you, where you work, what activities you are involved in, etc.*
2. What do you think is required for this transition to go smoothly? What are the key issues you think need to be addressed and by who?

*Ask:* Are there any documents you can share with me about that?

*We have reached the end of the interview. Is there anything else you would like to say, or do you have any questions for me?*

*Thank you for your time and participation today.*
